# Early complications and long-term outcomes of patients treated with a subcutaneous cardioverter-defibrillator: temporal trends and clinical implications of the anaesthetic strategies adopted at implant

**DOI:** 10.1093/europace/euag148

**Published:** 2026-06-12

**Authors:** Luca Donisi, Fawzi Kerkouri, Christelle Marquié, Vincent Probst, Vincent Algalarrondo, Alaa Al Amoura, Marc Badoz, Emilie Bastard, Nathalie Behar, Géraldine Bertaux, Francis Bessière, Pierre Bordachar, Abdeslam Bouzeman, Gilles Cellarier, Jean-Pierre Chabert, Laure Champ-Rigot, Gaël Clerici, Antoine Da Costa, Pascal Defaye, Jean-Claude Deharo, Camille Delahaye, Christian Demasles, Romain Eschalier, Laurent Fauchier, Denis Gaty, Hervé Gorka, Cyril Goujeau, Charles Guenancia, Aurélie Guiot, Alexis Hermida, Isabelle Heurtebise, Jérôme Hourdain, Peggy Jacon, Laurence Jesel, François Jourda, Mikael Laredo, Alain Lebon, Christophe Leclercq, François Lesaffre, Jean Litalien, Christophe Loose, Vincent Mansourati, Philippe Maury, Alexis Mechulan, Antoine Milhem, Aurélien Miralles, Frédérique Mizon-Gérard, Pierre Mondoly, Ghassan Moubarak, Jean-Luc Pasquié, David Perrot, Mickaël Peyrol, Anne Quentin, Nicolas Sadoul, Marc Strik, Claire Vannesson, Frédéric Victor, Aude Zanutto, Serge Boveda, Eloi Marijon, Rodrigue Garcia

**Affiliations:** Division of Cardiology, European Georges Pompidou Hospital, Paris, France; Université Paris Cité, PARCC, INSERM U970, Paris, France; Department of Clinical Sciences and Community Health, University of Milan, Milan, Italy; Division of Cardiology, European Georges Pompidou Hospital, Paris, France; Université Paris Cité, PARCC, INSERM U970, Paris, France; Univ Brest, Laboratoire ORPHY EA 4324, Brest F-29200, France; Department of Cardiology, University Hospital of Brest, Brest, France; Department of Cardiology, University Hospital of Lille, Lille, France; Department of Cardiology, University Hospital of Nantes, Nantes, France; Department of Cardiology, Bichat Hospital, Paris, France; Department of Cardiology, Centre Hospitalier de Troyes, Troyes, France; Department of Cardiology, University Hospital of Besancon, Besancon, France; Department of Cardiology, Polyclinique Les Fleurs, Toulon, France; Department of Cardiology, Hopital Pontchaillou, Rennes, France; Department of Cardiology, CHU Dijon Bourgogne, Dijon, France; Department of Cardiology, Hospices Civils de Lyon, Lyon, France; Department of Cardiology, University Hospital of Bordeaux, Bordeaux, France; Department of Cardiology, Hopital privé de Parly 2, Le Chesnay-Rocquencourt, France; Department of Cardiology, Hopital d’instruction des armées Sainte-Anne, Toulon, France; Department of Cardiology, CHU de Reims, Reims, France; Department of Cardiology, University Hospital of Caen, Caen, France; Department of Cardiology, University Hospital of La Réunion, Saint-Pierre, La Réunion, France; Department of Cardiology, University Hospital of Saint-Etienne, Saint-Etienne, France; Department of Cardiology, University Hospital of Grenoble, Grenoble, France; Department of Cardiology, University Hospital of Marseille - La Timone, Marseille, France; Department of Cardiology, Centre Hospitalier de Roubaix, Roubaix, France; Department of Cardiology, Centre Hospitalier de Bigorre, Tarbes, France; Department of Cardiology, University Hospital of Clermont-Ferrand, Clermont-Ferrand, France; Department of Cardiology, University Hospital of Tours, Tours, France; Department of Cardiology, Centre Hospitalier de Carcassonne, Carcassonne, France; Department of Cardiology, Centre Hospitalier de Chartres, Chartres, France; Department of Cardiology, Centre Hospitalier de Saintonge, Saintes, France; Department of Cardiology, CHU Dijon Bourgogne, Dijon, France; Department of Cardiology, Hôpital privé Bois Bernard, Lens, France; Department of Cardiology, CHU Amiens, Amiens, France; Department of Cardiology, Centre Hospitalier Jacques Cœur, Bourges, France; Department of Cardiology, University Hospital of Marseille - La Timone, Marseille, France; Department of Cardiology, University Hospital of Grenoble, Grenoble, France; Department of Cardiology, University Hospital of Strasbourg, Strasbourg, France; Department of Cardiology, Centre Hospitalier d’Auxerre, Auxerre, France; Department of Cardiology, La Pitié-Salpêtrière Hospital, Paris, France; Department of Cardiology, Hôpital privé Saint-Martin, Caen, France; Department of Cardiology, Hopital Pontchaillou, Rennes, France; Department of Cardiology, CHU de Reims, Reims, France; Department of Cardiology, Centre Hospitalier de Périgueux, Périgueux, France; Department of Cardiology, Clinique Saint-Gatien, Tours, France; Department of Cardiology, University Hospital of Brest, Brest, France; Department of Cardiology, University Hospital of Toulouse, Toulouse, France; Department of Cardiology, Hôpital privé Clairval, Marseille, France; Department of Cardiology, Centre Hospitalier de La Rochelle, La Rochelle, France; Department of Cardiology, Centre Hospitalier de Valence, Valence, France; Department of Cardiology, Hôpital privé Le Bois, Lille Metropole, France; Department of Cardiology, University Hospital of Toulouse, Toulouse, France; Department of Cardiology, Clinique Ambroise Paré, Neuilly-sur-Seine, France; Department of Cardiology, University Hospital of Montpellier, Montpellier, France; Division of Cardiology, European Georges Pompidou Hospital, Paris, France; Department of Cardiology, Hopital Nord, University Hospital of Marseille, Marseille, France; Department of Cardiology, Centre Hospitalier de Saint-Brieuc, Saint-Brieuc, France; Department of Cardiology, University Hospital of Nancy, Nancy, France; Department of Cardiology, University Hospital of Bordeaux, Bordeaux, France; Department of Cardiology, Centre Hospitalier de Lens, Lens, France; Department of Cardiology, Polyclinique Saint Victor, Rennes, France; Department of Cardiology, CHR Mercy, Metz, France; Department of Cardiology, Pasteur Clinic, Toulouse, France; Brussels University, VUB, Brussels, Belgium; Division of Cardiology, European Georges Pompidou Hospital, Paris, France; Université Paris Cité, PARCC, INSERM U970, Paris, France; Department of Cardiology, University Hospital of Poitiers, Poitiers, France; Centre d’Investigation Clinique 1402, University Hospital of Poitiers, Poitiers, France

**Keywords:** General anaesthesia, Resources consumption, Subcutaneous implantable cardioverter defibrillator (S-ICD), Sudden cardiac death, Complications, Mortality

## Abstract

**Aims:**

Subcutaneous implantable cardioverter defibrillator (S-ICD) therapy is a well-established therapy for the prevention of sudden cardiac death. Although general anaesthesia (GA) was initially advocated for implantation, non-GA has emerged as a feasible alternative in clinical practice. This study evaluated the early and long-term outcomes associated with anaesthetic modality during S-ICD implantation procedures.

**Methods and results:**

The nationwide, single-arm, observational HONEST cohort study included all patients implanted with an S-ICD (EMBLEM™, Boston Scientific) in France between 2012 and 2019. GA was characterised by controlled unconsciousness with airway management, while non-GA included all alternative techniques. Among 4924 patients, 1041 (21.1%) underwent non-GA. The proportion of non-GA use increased from 2.5% to 26.9% between 2012 and 2019 (*P* < 0.001). Patients undergoing implantation under non-GA tended to be older (51 ± 14 vs. 50 ± 15 years), had lower left ventricular ejection fraction (41 ± 16 vs. 43 ± 17%), and fewer secondary prevention indications (33 vs. 38%) (all *P* < 0.01). The 30-day complication rate was 3.4% in the non-GA group (vs. 3.7% in the GA group; *P* = 0.85). At follow-up, the incidence of overall complications (4.29 events per 100 person-years for non-GA vs. 4.73 for GA; *P* = 0.937), reintervention (1.21 vs. 1.62; *P* = 0.963), inappropriate shocks (2.88 vs. 2.78; *P* = 0.782), and all-cause mortality (2.93 vs. 2.56; *P* = 0.938) were similar between groups. Multivariable analysis confirmed that there were no differences across outcomes (all *P* values >0.1).

**Conclusion:**

The anaesthesia strategy used for S-ICD implantation was not associated with poorer short- or long-term clinical outcomes, supporting non-general anaesthesia as a feasible alternative in appropriately selected patients.

**ClinicalTrials.gov ID:**

NCT05302115

## Graphical Abstract

**Graphical Abstract:**
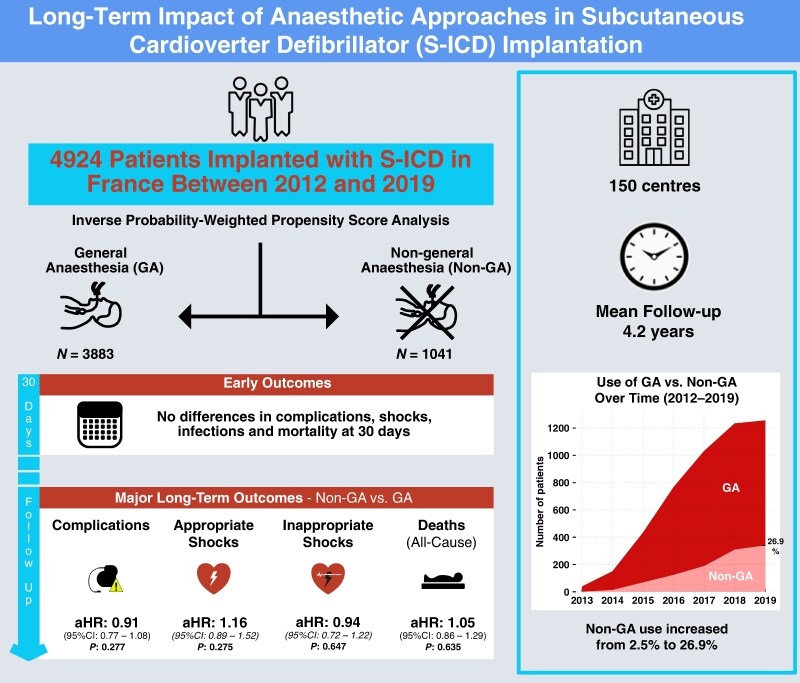
Perioperative and long-term outcomes related to anaesthetic modality in subcutaneous implantable cardioverter defibrillator implantation—a propensity score-weighted analysis from the HONEST nationwide cohort.

What’s new?

- This is the largest nationwide study to date comparing general anaesthesia (GA) vs. non-general anaesthesia (non-GA) strategies for subcutaneous implantable cardioverter-defibrillator (S-ICD) implantation.
- Non-GA use increased more than tenfold in France between 2012 and 2019, reflecting a major shift in clinical practice.
- Early and long-term outcomes—including complications, reinterventions, appropriate and inappropriate shocks, and mortality—were comparable between GA and non-GA after a mean follow-up of 4 years.

## Introduction

The subcutaneous implantable cardioverter-defibrillator (S-ICD) has gained widespread adoption as a viable alternative to transvenous implantable cardioverter-defibrillators for the prevention of sudden cardiac death (SCD) in patients who do not require cardiac pacing.^1–3^ However, there are notable differences in the anaesthetic management of S-ICD and transvenous cardioverter-defibrillator implantation. Despite increasing experience with S-ICD implantation, general anaesthesia remains the most utilized approach, while non-general anaesthesia techniques are used in only a minority of cases, ranging from 1% to 14% according to recent national and European surveys.^4,5^

The initial reluctance to use non-GA for S-ICD procedures likely reflects concerns related to the device's larger size, more extensive tissue dissection, lead tunnelling, and mandatory defibrillation testing, all of which contribute to the procedure’s invasive and painful nature. Nevertheless, as operator experience grows, S-ICD implantation continues to expand worldwide.^6,7^ Recent studies have shown that non-GA approaches are safe, more cost-effective, and associated with reduced procedural times and lower post-procedural pain compared with GA.^8–12^ Furthermore, a secondary analysis of the PRAETORIAN-DFT trial found no significant differences in pain perception between patients implanted under GA or non-GA.^13^ While recent data have shown comparable clinical endpoints between general anaesthesia and deep sedation,^14^ large-scale, real-world studies assessing patient characteristics and long-term clinical outcomes according to anaesthesia strategy remain limited. This gap in knowledge is particularly relevant given the absence of clear guideline recommendations regarding the preferred anaesthetic approach for S-ICD implantation, leaving the choice largely to operator discretion and patient preference.^15^

In this context, our study endeavoured to address this gap by comprehensively comparing GA and non-GA during S-ICD implantation, not only regarding peri-procedural outcomes but also long-term clinical endpoints. Using a large nationwide dataset, we aimed to provide robust evidence to guide future clinical practice regarding the optimal anaesthetic strategy for S-ICD implantation.

## Methods

### Study design and participants

We analysed data from 4924 patients enrolled in the ongoing HONEST Study (NCT05302115). Briefly, the HONEST cohort (coHOrte fraNçaise des dEfibrillateurs Sous cuTanés) is a nationwide, academic, single-arm, observational study conducted in France, encompassing all patients implanted with a subcutaneous implantable cardioverter-defibrillator (S-ICD, EMBLEM, Boston Scientific) between October 2012 (the date of the first S-ICD implantation in France) and December 2019, across all 150 French hospitals accredited for S-ICD implantation.^16–19^ These institutions include 39 university hospitals, 60 non-university teaching hospitals, and 51 private centres. To achieve comprehensive patient inclusion, serial numbers of S-ICD generators invoiced for the French market by Boston Scientific were used, corresponding to 5175 devices. Of these, 5053 (99.4%) were included: 4924 *de novo* implants and 129 generator replacements. The remaining devices were either in pharmacy stocks (31) or linked to patients who declined participation (91) (see Supplementary material online, *Figure S1*). The cohort included predominantly adult patients; 100 patients received S-ICD implantation before the age of 18 years but were followed and consented as adults at the time of data collection. Baseline, procedural, and follow-up data were retrospectively collected between 2018 and 2019, and prospective annual follow-up was initiated in 2020. Patient consent was obtained from all participants, and data management was centralized at the Paris Cardiovascular Research Centre-INSERM U970 to ensure data integrity and security. The study adhered to the principles of the Helsinki Declaration and received approval from the French Data Protection Committee (CNIL, registry no. 2217196).

### Anaesthetic techniques

Anaesthetic techniques were defined according to the U.S. expert panel consensus on S-ICD anaesthesia.^20^ GA was defined as the administration of intravenous or inhaled anaesthetic agents to induce controlled unconsciousness, requiring airway management via endotracheal intubation or a supraglottic device. Patients under GA were continuously monitored for haemodynamic stability and received intra-operative analgesia according to institutional protocols. Non-general anaesthesia (non-GA) was defined as the use of one of the following two techniques: (i) Monitored Anaesthesia Care (MAC), referring to the administration of local anaesthesia at the generator and tunnelling sites, combined with moderate-to-deep sedation. Sedation is provided and continuously managed by an anaesthesia professional (anaesthesiologist or nurse anaesthetist) who monitors vital signs and ensures patient stability. (ii) Non-Anaesthesia Personnel Administered Sedation and Analgesia (NASA): sedation administered by non-anaesthesia-trained healthcare providers, such as electrophysiologists or nurses. Sedation under NASA protocols is typically lighter, adheres to strict procedural guidelines, and aims to maintain patient comfort while minimizing the need for deep sedation.^8,9,13,21,22^ Because ‘non-GA’ may include local anaesthesia alone or local anaesthesia with sedation, we classified anaesthesia strategy primarily according to the level of anaesthesia involvement and supervision. The NASA subgroup denotes procedures performed without anaesthesia-team supervision. Detailed agent-level sedative/analgesic data were not available with sufficient completeness for robust comparative analyses. Thoracic regional blocks were performed in 76 patients (7.3%). All thoracic blocks were performed by the implanting cardiologists (not the anaesthesia team). Given the limited number of cases and the lack of standardised protocols, these patients were included in the non-general anaesthesia group and analysed within the NASA subgroup. The choice between GA and non-GA was based on shared decision-making, the availability of anaesthesia services, and operator preference.

### Clinical outcomes and endpoints

Baseline characteristics and procedural data were collected from the electronic medical records. All clinical events, including death, complications, and device therapies (appropriate and inappropriate shocks), were independently reviewed and adjudicated by the study investigators according to pre-defined criteria. Appropriate and inappropriate shocks were recorded through regular clinical follow-up and remote monitoring using the Latitude^TM^ system, which covered 96% of the patients, ensuring high-quality longitudinal data. Vital status was verified using the French National Institute of Statistics and Economic Studies (INSEE) database, which comprehensively tracks mortality across France. Investigating sites adjudicated and reported S-ICD shocks and related complications directly into the database. To ensure consistency and accuracy, an expert adjudication committee conducted centralized reviews of all S-ICD-related deaths, infections, and shock episodes (see Supplementary material online, *Table S11*). In addition, an external data quality audit conducted in 2023 in a large sample of the cohort (2966 patients; 60.2%) confirmed high data completeness with minimal discrepancies (see Supplementary material online, *Table S12*).

The primary outcomes of interest were early complications (occurring within 30 days after S-ICD implantation) and overall complications observed throughout the follow-up period. In addition, the occurrence of appropriate and inappropriate shocks was assessed alongside all-cause mortality and cause-specific mortality. Appropriate shocks were defined as therapies delivered in response to ventricular arrhythmias exceeding the programmed detection thresholds, whereas inappropriate shocks were defined as those delivered for non-ventricular arrhythmias, cardiac or extracardiac oversensing, or ventricular arrhythmias below the programmed threshold. Local complications included pocket haematoma, infection, poor wound healing, generator migration, and device externalization. Infections were further classified as local or systemic according to the modified Duke criteria. Lead-related complications were defined as lead dislodgement, fracture, or issues related to signal detection or impedance. Deaths were categorized according to the European Society of Cardiology guidelines into cardiovascular, non-cardiovascular, S-ICD-related, or unknown causes, with precise documentation of the primary cause of death.^23^ S-ICD-related deaths were defined as fatalities directly attributable to device implantation or replacement or deaths that would not have occurred in the absence of device-related complications.

### Statistical analysis

Categorical variables were summarized as counts and percentages, and continuous variables were reported as mean ± standard deviation. Incidence density was computed to account for differences in follow-up duration. Group comparisons were conducted using χ^2^ or Fisher’s exact tests for categorical variables and Student’s *t*-test or Wilcoxon rank-sum tests for continuous variables, as appropriate.

The primary analysis compared the outcomes between patients who underwent GA and those who underwent non-GA at the time of implantation. Additionally, subgroup analyses were performed to evaluate outcomes across the two non-GA techniques (NASA and MAC) to explore whether specific strategies within the non-GA category were associated with differential outcomes.

The associations between anaesthesia type and outcomes were evaluated using Cox proportional hazards models, with standard errors adjusted for centre effects. Crude analyses provided unadjusted hazard ratios (HRs), while adjusted HRs were derived from weighted populations following propensity score (PS) analysis using inverse probability weighting (IPW) to account for baseline differences. IPW weights were constructed to estimate the average treatment effect (ATE).

Baseline characteristics, event rates, and adjusted associations between anaesthetic modality (NASA vs. MAC) and outcomes were evaluated using the same statistical methods and covariate adjustment strategy applied to the primary GA vs. non-GA analyses. Results are reported in Supplementary material online, *Tables S1–S3*. Associations between anaesthesia strategy and time-to-event outcomes were evaluated using Cox proportional hazards models including a centre-level random effect (shared frailty term) to account for between-centre heterogeneity and within-centre clustering.

To evaluate the impact of centre effects, we performed sensitivity analyses incorporating centre status (academic vs. non-academic) and centre volume; neither was significantly associated with outcomes.

To mitigate residual confounding, implant year and generator position (subcutaneous vs. intermuscular) have been added to the propensity score model. The Love plots (see Supplementary material online, *Figures S2*–*S7*) document covariate balance.

Recurrent shock events were analysed using Andersen-Gill models with robust variance estimators, while complications and complications requiring reintervention were analysed using the Wei, Lin, and Weissfeld model.

For all outcomes, follow-up was censored at device removal, loss to follow-up, or end of study. A total of 446 patients (9.1%) were lost to follow-up and were treated as missing data at random. Because loss-to-follow-up rates differed modestly between anaesthesia groups, anaesthesia strategy and all baseline covariates were included in the imputation model, and sensitivity analyses restricted to patients with complete follow-up yielded results consistent with the primary analysis. Baseline characteristics of patients lost to follow-up vs. retained are shown in Supplementary material online, *Table S4*, supporting the plausibility of a Missing At Random mechanism.

All statistical tests were two-sided, and a *P*-value of <0.05 was considered statistically significant. Analyses were performed using R software version 4.4.1.

## Results

### Anaesthesia modality and temporal trends

The HONEST cohort included 4924 patients (mean age 50.0 ± 15.0 years) who underwent S-ICD implantation. Among them, 3883 (78.9%) received GA and 1041 (21.1%) received non-GA. Within the non-GA group, 551 patients (52.9%) underwent MAC, and 490 patients (47%) received NASA.

**Figure 1:**
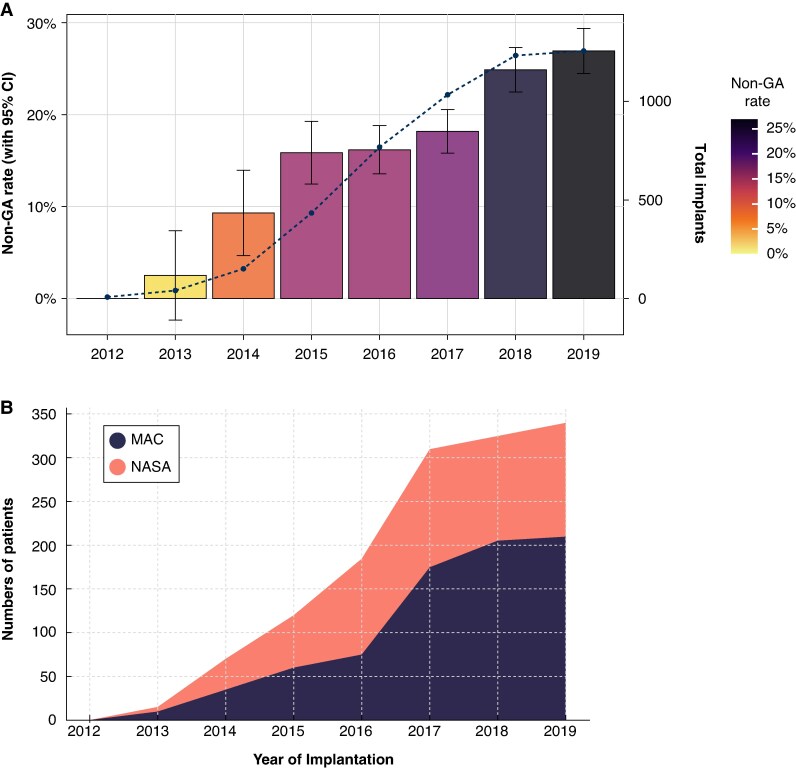
Temporal trends in non-general anaesthesia use: (*A*) overall non-general anaesthesia (non-GA) utilization and (*B*) technique breakdown—MAC vs. NASA.

The use of non-GA increased significantly over time, from 2.5% to 26.9% between 2013 and 2019 (*P* < 0.001), with notable surges between 2013 and 2015 (up to 15.9%) and 2017 and 2018 (from 18.2% to 24.9%). Among the different non-GA modalities, the adoption of MAC increased most significantly during the study period (*P* for trend <0.001) (*Figure 1*).

### Baseline characteristics and perioperative complications

The baseline and procedural characteristics of the anaesthesia groups are summarized in *Table 1*. Compared with patients who underwent S-ICD implantation using GA, those managed with non-GA were less likely to be female (20.6% vs. 24.1%, *P* = 0.018), more frequently implanted for the primary prevention of SCD (67.0% vs. 62.4%, *P* = 0.006), and had a higher prevalence of structural heart disease (80.7% vs. 77.5%, *P* = 0.029). Left ventricular ejection fraction was 40.8 ± 16.2% in the non-GA group (vs. 42.7 ± 16.9% in the GA group; *P* = 0.002). Procedural practices also differed; two-incision technique was more common among non-GA patients (96.7% vs. 87.9%, *P* < 0.001), and defibrillation testing was less frequently performed (61.8% vs. 88.2%, *P* < 0.001).

**Table 1:** Baseline characteristics of the population according to the performance of non-GA or GA at implantation

Characteristic^a^	Non-GA *n* = 1041	GA *n* = 3883	*P*-value
Age, *years*	51.4 ± 14.2	49.5 ± 15.2	<0.001
Female Sex	214 (20.6%)	934 (24.1%)	0.018
BMI^b^, *kg/m*^2^	26.7 ± 8.3	26.2 ± 5.6	0.235
Diabetes^c^	168 (17.4%)	509 (14.9%)	0.051
S-ICD indication			0.006
*Primary prevention*	697 (67.0%)	2422 (62.4%)	
*Secondary prevention*	344 (33.0%)	1461 (37.6%)	
Type of Secondary Prevention			0.011
Cardiac arrest	257 (74.7%)	978 (66.9%)	
Sustained ventricular tachycardia	69 (20.1%)	354 (24.2%)	
Syncope and inducible VT at EPS	18 (5.2%)	129 (8.8%)	
Underlying heart disease			0.029
*Structural heart disease*	840 (80.7%)	3011 (77.5%)	
*Electrical heart disease*	201 (19.3%)	872 (22.5%)	
LVEF^d^, *%*	40.8 ± 16.2	42.7 ± 16.9	0.002
History of ICD implantation	103 (9.9%)	548 (14.1%)	<0.001
Associated pacing system	14 (1.3%)	68 (1.8%)	0.363
Defibrillation testing	643 (61.8%)	3423 (88.2%)	<0.001
Type of generator^d^			<0.001
1st generation 1010	28 (2.7%)	286 (7.4%)	
2nd and 3rd generation A209/A219	1013 (97.3%)	3595 (92.6%)	
Number of incisions			<0.001
*2*	1007 (96.7%)	3413 (87.9%)	
*3*	34 (3.3%)	470 (12.1%)	
Subcutaneous position	458 (44.0%)	1743 (44.9%)	0.632

^a^Data are mean ± sd or *n* (%).

^b^Missing data: 118 for non-GA, 860 for GA.

^c^Missing data: 77 for non-GA, 456 for GA.

^d^Missing data: 2 for GA.

Abbreviations: EPS, electrophysiology study; GA, general anaesthesia; ICD, implantable cardioverter defibrillator; S-ICD, subcutaneous implantable cardioverter defibrillator; VT, ventricular tachycardia.

Crude analyses of the perioperative period demonstrated similar results between the groups. Thirty days after S-ICD implantation, the complication rate was 3.4% (95% CI: 2.3–4.5%) in the non-GA group and 3.7% (95% CI: 3.1–4.3%) in the GA group. The incidence of inappropriate shocks was 0.9% (95% CI: 0.3–1.4%) in the non-GA group and 1.2% (95% CI: 0.9–1.5%) in the GA group. Other perioperative complications, including local complications (2.2% vs. 2.1%), infections (0.5% vs. 0.8%), pocket haematoma (1.1% vs. 0.9%), lead complications (<0.1% in both groups), chronic device-related pain (<0.1% in both groups), complications requiring intervention (0.9% vs. 0.7%), and all-cause mortality (0.2% in both groups), were similar in both groups.

### Mid- and long-term outcomes

**Figure 2:**
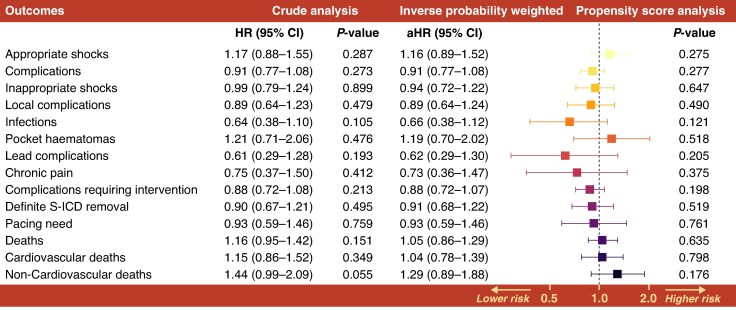
Forest plots of outcomes in patients implanted with non-GA compared with those implanted with GA.

**Figure 3:**
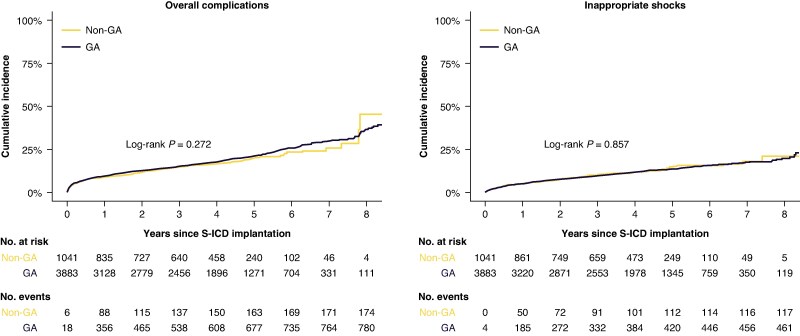
Cumulative incidence of overall complications and inappropriate shocks: non-GA vs. GA.

The mean follow-up duration was slightly shorter in the non-GA group compared with the GA group (3.9 ± 1.9 vs. 4.3 ± 2.2 years; *P* < 0.001). As shown in *Table 2* and *Figures 2* and *3*, overall complication rates were similar between groups (4.29 vs. 4.73 events per 100 person-years for non-GA and GA, respectively; rate difference −0.45; *P* = 0.937). IPW confirmed the absence of significant differences [adjusted hazard ratio (aHR) 0.91; 95% CI: 0.77–1.08; *P* = 0.277].

**Table 2:** Clinical outcomes in patients implanted with non-GA and GA

Event	Non-GA; *n* = 1041	GA; *n* = 3883	Rate Difference	*P*-value
Overall Complications	4.29	4.73	−0.45	0.937
Inappropriate shocks	2.88	2.78	0.10	0.782
Local complications	1.08	1.19	−0.11	0.869
*Infection*	0.39	0.58	−0.19	0.952
*Pocket haematoma*	0.44	0.37	0.07	0.878
*Poor wound healing*	0.27	0.28	−0.01	0.443
*Other*	0.07	0.06	0.01	0.750
Lead complications	0.2	0.36	−0.16	0.963
*Lead dislodgement*	0.1	0.18	−0.08	0.944
*Lead fracture*	0.05	0.09	−0.04	0.920
*Lead noise*	0.02	0.04	−0.02	0.871
Chronic pain	0.22	0.34	−0.12	0.944
Complications Requiring Intervention	1.21	1.62	−0.41	0.963
Definite S-ICD removal	1.9	1.86	0.04	0.578
Pacing need	0.57	0.67	−0.10	0.901
Appropriate shocks	3.25	2.79	0.46	0.948
Death	2.93	2.56	0.37	0.938
Cardiovascular	1.5	1.33	0.17	0.905
Non-Cardiovascular	0.91	0.65	0.26	0.953
S-ICD-related	0.02	0.04	−0.02	0.870
Unknown	0.49	0.55	−0.05	0.821

Incidence density is in number of events per 100 person-years.

Abbreviations: GA, general anaesthesia.

Inappropriate shock rates were also comparable (2.88 vs. 2.78 events per 100 person-years for non-GA and GA; rate difference +0.10; *P* = 0.782), with adjusted analysis showing no significant difference (aHR 0.94; 95% CI: 0.72–1.22; *P* = 0.647).

The incidence of complications requiring intervention was similar across groups (1.21 vs.1.62 events per 100 person-years for non-GA and GA; rate difference −0.41; *P* = 0.963). IPW-adjusted analyses yielded consistent results: complications requiring intervention (aHR 0.88; 95% CI: 0.72–1.07; *P* = 0.198), local complications (aHR 0.89; 95% CI: (0.64–1.24); *P* = 0.490), and lead-related complications (aHR 0.62; 95% CI: 0.29–1.30; *P* = 0.205). Subgroup analyses stratified by age (≥70 vs. <70 years), sex, LVEF (≤35%), and diabetes status showed no significant differences between GA and non-GA (see Supplementary material online, *Tables S5*–*S10*).

Appropriate shock rates were similar between groups (3.25 vs. 2.79 events per 100 person-years for non-GA and GA; rate difference +0.46; *P* = 0.948), with no significant difference after adjustment (aHR 1.16; 95% CI: 0.89–1.52; *P* = 0.275).

All-cause mortality was comparable (2.93 vs. 2.56 events per 100 person-years for non-GA and GA; rate difference +0.37; *P* = 0.938), with IPW-adjusted HR of 1.05 (95% CI: 0.86–1.29; *P* = 0.635). Cardiovascular mortality was also similar (1.5 vs.1.33 events per 100 person-years for non-GA and GA; rate difference +0.17; *P* = 0.905), while non-cardiovascular mortality was slightly higher in the non-GA group (0.91 vs. 0.65 events per 100 person-years; rate difference +0.26; *P* = 0.953). Prespecified subgroup analyses stratified by sex, age, left ventricular ejection fraction, and diabetes mellitus showed consistent results across all groups, with no evidence of treatment-effect heterogeneity between GA and non-GA strategies (see Supplementary material online, *Tables S5*–S10).

## Non-general anaesthesia modality: MAC vs. NASA

A subgroup analysis was conducted within the non-GA cohort to assess whether the anaesthetic management approach influenced clinical outcomes. Among 1041 patients with non-GA, 551 (53%) patients received MAC, and 490 (47%) received NASA.

Compared with the MAC group, patients in the NASA group were significantly younger (49.7 ± 15.2 vs. 52.8 ± 13.1 years; *P* < 0.001), had a higher prevalence of electrical heart disease (25.9% vs. 13.4%; *P* < 0.001), and higher left ventricular ejection fraction (44.4 ± 16.4% vs. 37.5 ± 15.3%; *P* < 0.001). Moreover, defibrillation testing was less frequently performed in the NASA group (54.5% vs. 68.2%; *P* < 0.001), with 2-incision technique utilized in most patients (98.8% vs. 94.9% for NASA and MAC respectively, *P*-value <0.001) and follow-up duration slightly longer (4.05 ± 1.9 vs. 3.7 ± 1.9 years; *P* = 0.033) (see Supplementary material online, *Table S1*). Event rates are reported in Supplementary material online, *Table S2*, however, after adjustment, multivariable analyses revealed no statistically significant differences between groups in the risk of overall complications (HR 1.26; 95% CI: 0.92–1.73; *P* = 0.148), inappropriate shocks (HR 1.28; 95% CI: 0.83–1.99; *P* = 0.268) and appropriate shocks (HR 0.84; 95% CI: 0.52–1.36; *P* = 0.477) (see Supplementary material online, *Table S3*).

## Discussion

This prospective study offers the most extensive evaluation to date of anaesthetic strategies during S-ICD implantation, encompassing both perioperative and long-term outcomes. In contrast to earlier smaller studies focusing on single techniques or immediate procedural metrics, our analysis compared broad anaesthesia categories (GA vs. non-GA) in a large, real-world cohort with extended follow-up. The results suggest that non-GA approaches are a safe alternative to GA in both the short and long term. We observed no significant differences in overall complication rates, inappropriate shock incidence, lead-related issues, or reinterventions between the non-GA and GA groups, both early after implantation and throughout follow-up. Consistent with prior evidence, our sex-stratified analyses align with the i-SUSI project,^24^ which reported no increase in device-related complications among female compared with male S-ICD recipients.

Our data also highlight evolving practice patterns. Non-GA techniques were increasingly adopted over the study period, accounting for over one-quarter of S-ICD implants by 2019. This trend mirrors reports from other regions, pointing to a broader shift toward less invasive anaesthesia in device implantation. For example, a U.S. post-approval registry found that approximately one-third of S-ICD procedures were performed with conscious sedation or local anaesthesia.^5^ Nonetheless, GA remains the dominant practice in many centres, likely due to operator familiarity and the absence of definitive guidelines favouring sedation-based protocols. Our findings help fill this evidence gap. To our knowledge, this is the first prospective study directly comparing GA and non-GA anaesthesia in a large S-ICD cohort with hard clinical endpoints, and it affirms that foregoing GA was not associated with worse safety outcomes. This should encourage guideline committees and practitioners to consider non-GA approaches as a standard option, rather than reserving them for exceptional circumstances.

Because S-ICD therapy is frequently considered in younger patients, including those with inherited arrhythmia syndromes or congenital and structural substrates, optimizing procedural pathways has potential population-level relevance. Recent epidemiological analyses suggest divergent trends in SCD among young adults, with increases reported in some settings and persistent rates of unwitnessed events despite declining overall incidence, underscoring heterogeneity in prevention gaps.^25^ Against this background, anaesthesia strategy may have important health-system implications, as general anaesthesia availability and peri-procedural resource requirements can influence access, procedural throughput, and overall costs, in a context where premature cardiovascular mortality contributes materially to societal productivity losses.

Beyond clinical outcomes, the choice of anaesthesia has practical implications for procedural efficiency and resource utilization. General anaesthesia requires endotracheal intubation, dedicated anaesthesiology personnel, and prolonged post-anaesthesia recovery monitoring. These requirements translate into longer total time in the procedure room and greater consumption of hospital resources. International interpretation of ‘non-GA’ varies. In France, local infiltration anaesthesia is commonly used for cardiology procedures without anaesthesia-team involvement, with optional minimal-to-moderate sedation/analgesia (often benzodiazepines and/or opioids) depending on local practice. By contrast, propofol is an intravenous anaesthetic agent, usually administered by anaesthesia-trained physicians. Therefore, while we cannot adjudicate specific agents in our registry, the NASA category in this French cohort is unlikely to systematically reflect propofol-based deep sedation. Non-GA techniques offer the possibility to streamline the workflow: by obviating invasive airway management and deep sedation, non-GA has previously been associated with significantly shorter in-room and procedure times.^9^ Such time savings can increase procedural capacity, allowing more cases to be completed in a session and reducing scheduling backlogs. Moreover, eliminating or minimizing the need for an attending anaesthesiologist in the room (e.g. under moderate sedation protocols) may free anaesthesiology staff to cover multiple cases, addressing personnel constraints.

Non-GA approaches also appear to confer benefits in patient recovery, cost, and even environmental impact. Prior studies have shown that S-ICD implantation with local anaesthesia and light sedation results in lower intra- and post-operative pain scores and markedly reduced opioid requirements.^9,12^ Recently, De Veld et al. found no differences in post-operative pain between MAC and GA.^13^ Consistent with these reports, we found that patients in the non-GA group did not experience worse pain outcomes; in fact, chronic pain levels at 30 days and beyond were statistically indistinguishable from those in GA patients. Many high-volume centres now discharge the majority of S-ICD patients the same day of the procedure when using non-GA anaesthesia, with appropriate pain management protocols.^20,26^ By contrast, GA often necessitates longer observation or an overnight stay due to its systemic effects. Additionally, cost-efficiency is a notable advantage of non-GA strategies. A recent cost-effectiveness analysis demonstrated that using local anaesthesia in S-ICD implantation lowered total procedural costs by roughly 37% compared with GA.^8^ The savings were attributed to shorter operative times and avoidance of expensive anaesthetic medications and equipment, without any detriment to clinical outcomes. Finally, adopting non-GA approaches may diminish the environmental footprint of S-ICD procedures. Volatile anaesthetic gases used in GA, such as desflurane and sevoflurane, are potent greenhouse gases that substantially contribute to healthcare-related carbon emissions.^27^

While non-GA anaesthesia offers many advantages, it is not appropriate for every patient or setting, and our findings must be interpreted in context. Patients who are extremely anxious or unable to cooperate might still require GA for safety and comfort. Initial reservations about avoiding GA centred on the perceived difficulty of performing intra-operative S-ICD testing. Emerging evidence now shows that routine defibrillation testing can be safely omitted in most S-ICD recipients, substantially alleviating this concern.^17^ In this context, although DFT was less frequently performed in patients undergoing non-general anaesthesia, prior analyses from the HONEST cohort demonstrated no association between anaesthesia type and DFT failure;^17^ BMI values were relatively normal and did not differ between anaesthesia groups, suggesting that anaesthetic strategy was driven primarily by procedural characteristics and general anaesthesia availability, rather than body mass index.

Study limitations should be acknowledged. These findings primarily reflect adult S-ICD recipients and may not be directly generalizable to paediatric implantation, as children represented only a very small proportion of the implanted population.^28^ Moreover, despite the use of propensity score adjustment to mitigate baseline differences, this was not a randomised trial; the anaesthesia method was selected by the treating team, introducing potential selection bias. In addition, the long inclusion period captured evolving implantation techniques and learning-curve effects that could not be fully accounted for. Causal inferences are therefore limited. Second, although our cohort is the largest to date on this topic, some subgroups—particularly patients who received regional nerve blocks or tumescent anaesthesia—were relatively small and therefore not analysed. Third, our analysis did not collect data on specific anaesthetic complications or acute pain. Fourth, procedural duration was not systematically collected, and the potential contribution of defibrillation testing to procedure time could not be evaluated. Fifth, detailed information on specific anaesthetic agents, dosing, and supervision modality was not systematically collected in the registry. PRAETORIAN scoring could not be performed because standardised chest radiographs were unavailable and it was not systematically applied in patients in whom defibrillation testing was omitted, as the HONEST registry precedes its widespread clinical adoption.^29^ Finally, all implants were performed in France, and practice patterns (such as thresholds for same-day discharge or availability of anaesthesia support) may differ in other countries.^30^ Accordingly, confirmation of these findings in dedicated randomized controlled trials is warranted.

## Conclusion

In this large, prospective cohort study, non-GA was not associated with an increased risk of complications compared with GA. No significant differences were observed between non-GA and GA regarding perioperative or long-term outcomes, including complications, ICD therapies and mortality. Overall, the comparable safety profiles observed between non-general and general anaesthesia suggest that non-GA approaches may represent a feasible option in selected patients, while acknowledging that anaesthetic choice should remain individualized and that confirmation in randomized controlled trials is warranted.

## Supplementary Material

euag148_Supplementary_Data

## Data Availability

The data underlying this article will be shared on reasonable request to the corresponding author.
