# Supplementary material for: Early complications and long-term outcomes of patients treated with a subcutaneous cardioverter-defibrillator: temporal trends and clinical implications of the anaesthetic strategies adopted at implant
Source: Europace. 2026 Jun 12;28(7):euag148. doi: 10.1093/europace/euag148 (PMC13390802; doi:10.1093/europace/euag148)
Supplement: euag148_Supplementary_Data [file euag148_supplementary_data.docx]

**SUPPLEMENTAL MATERIAL**

**Supplemental Figure 1: Flow diagram depicting the derivation of the study population**

*
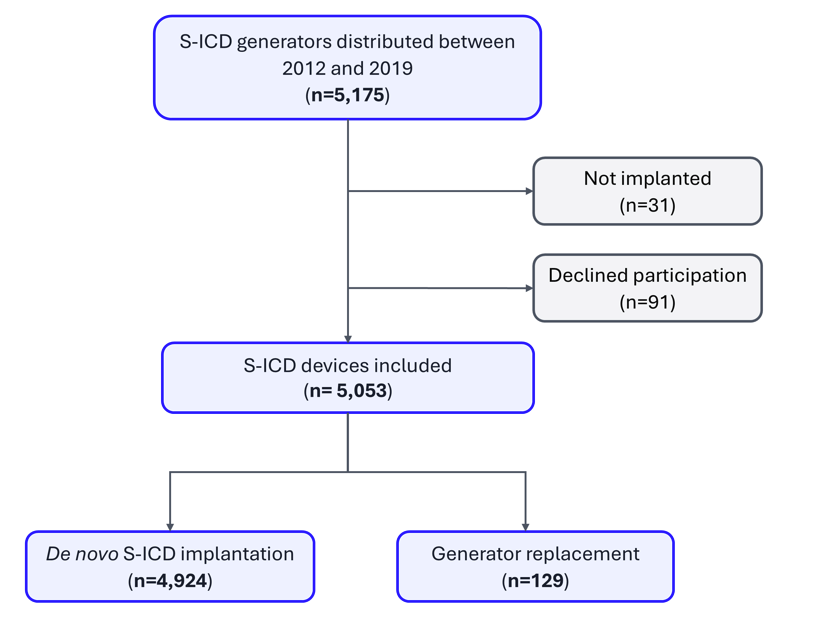
*

**Supplemental Figure 2: Loveplot before and after IPW PS for Complications analysis related to type of anesthesia (General vs Non general)**

*
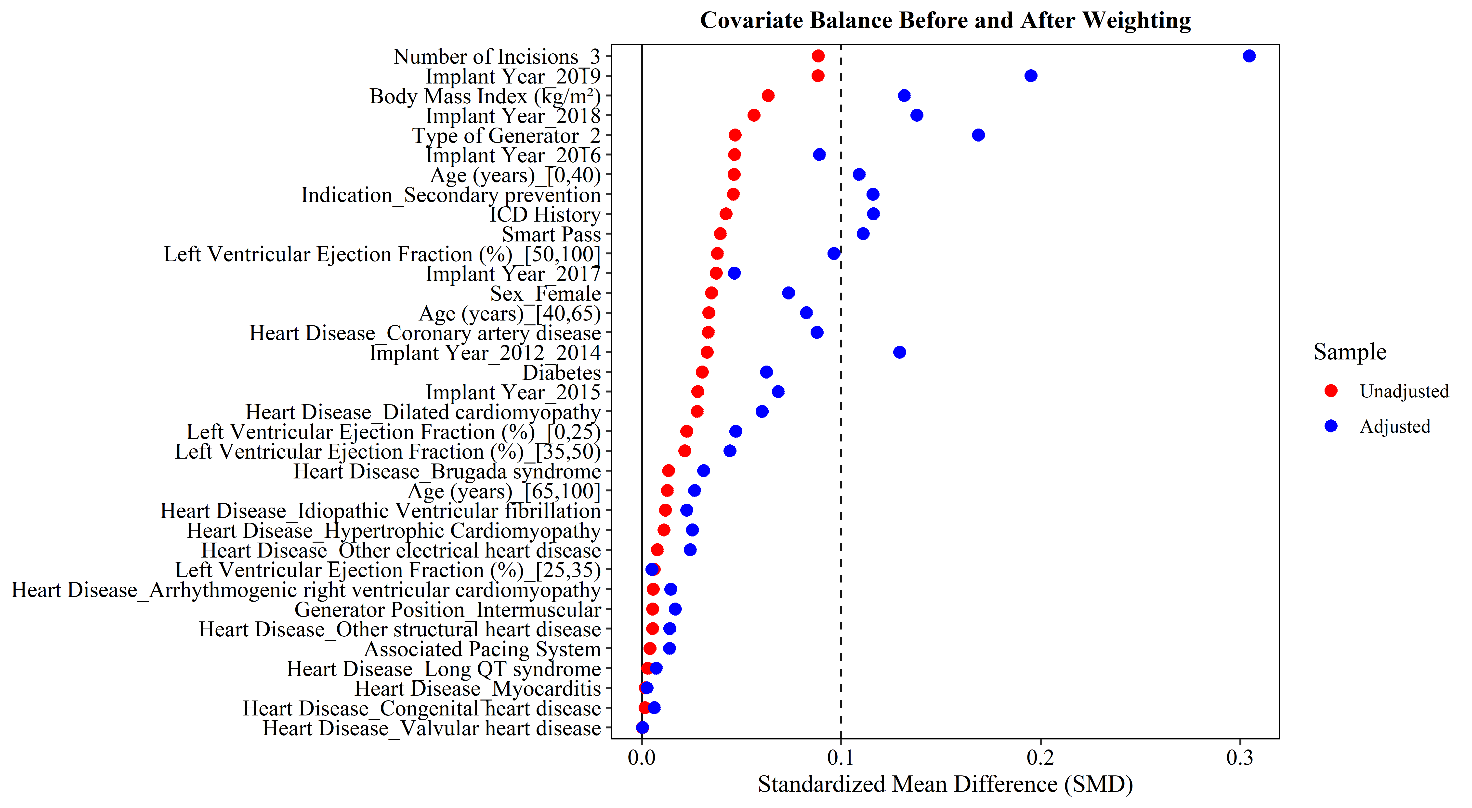
*

Variables included in the propensity score analysis are: age, sex, body mass index (bmi), diabetes, type of prevention (primary or secondary), underlying heart disease, left ventricular ejection fraction (LVEF), associated pacing (pacing system), history of tansvenous ICD, number of incisions (2 or 3), type of generator, defibrillator testing, and Smart Pass filter.

**Supplemental Figure 3: Loveplot before and after IPW PS for appropriate Shocks analysis related to type of anesthesia (General vs Non general)**

*
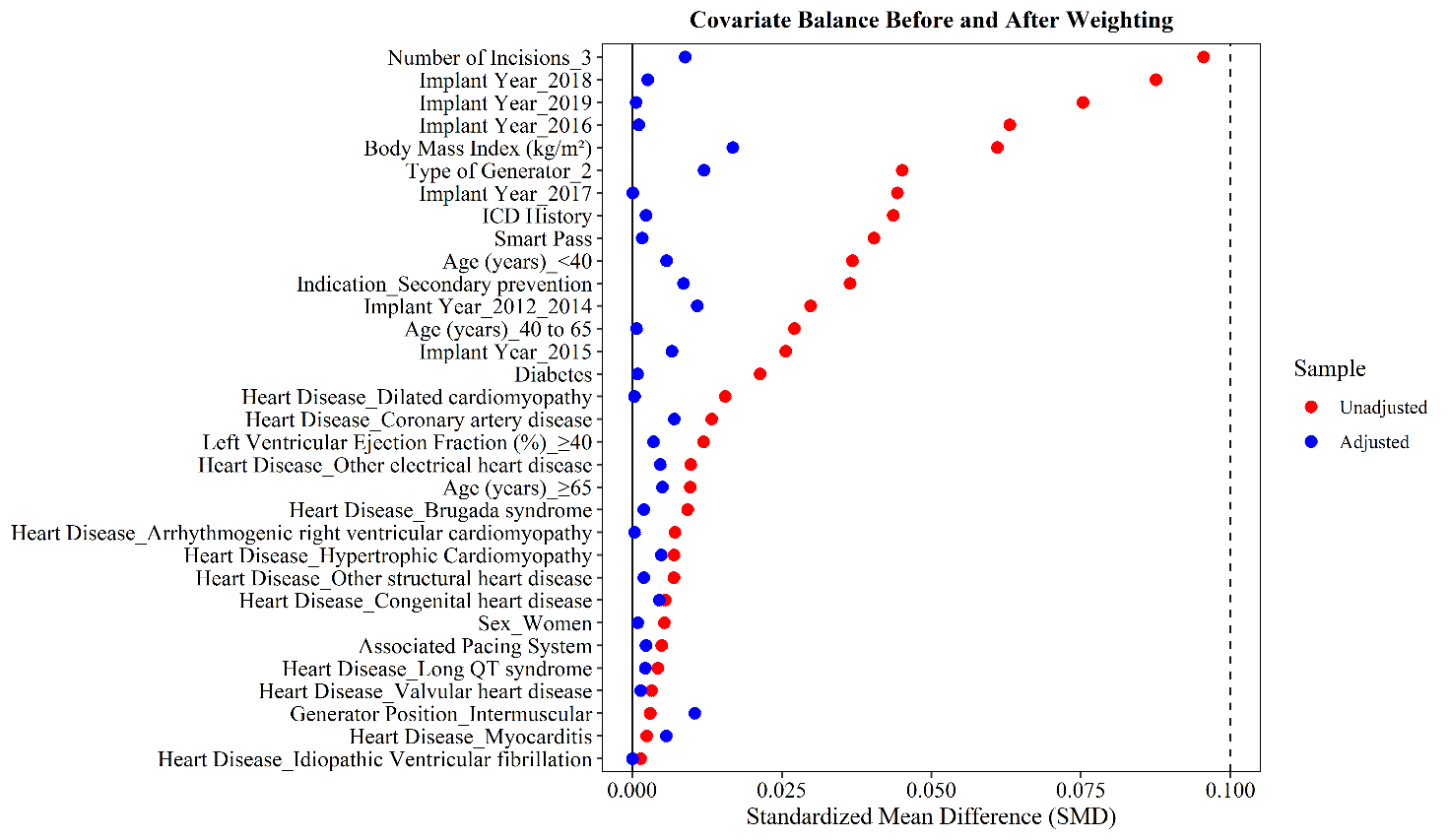
*

**Supplemental Figure 4: Loveplot before and after IPW PS for Inappropriate Shocks analysis related to type of anesthesia (General vs Non general)**

*
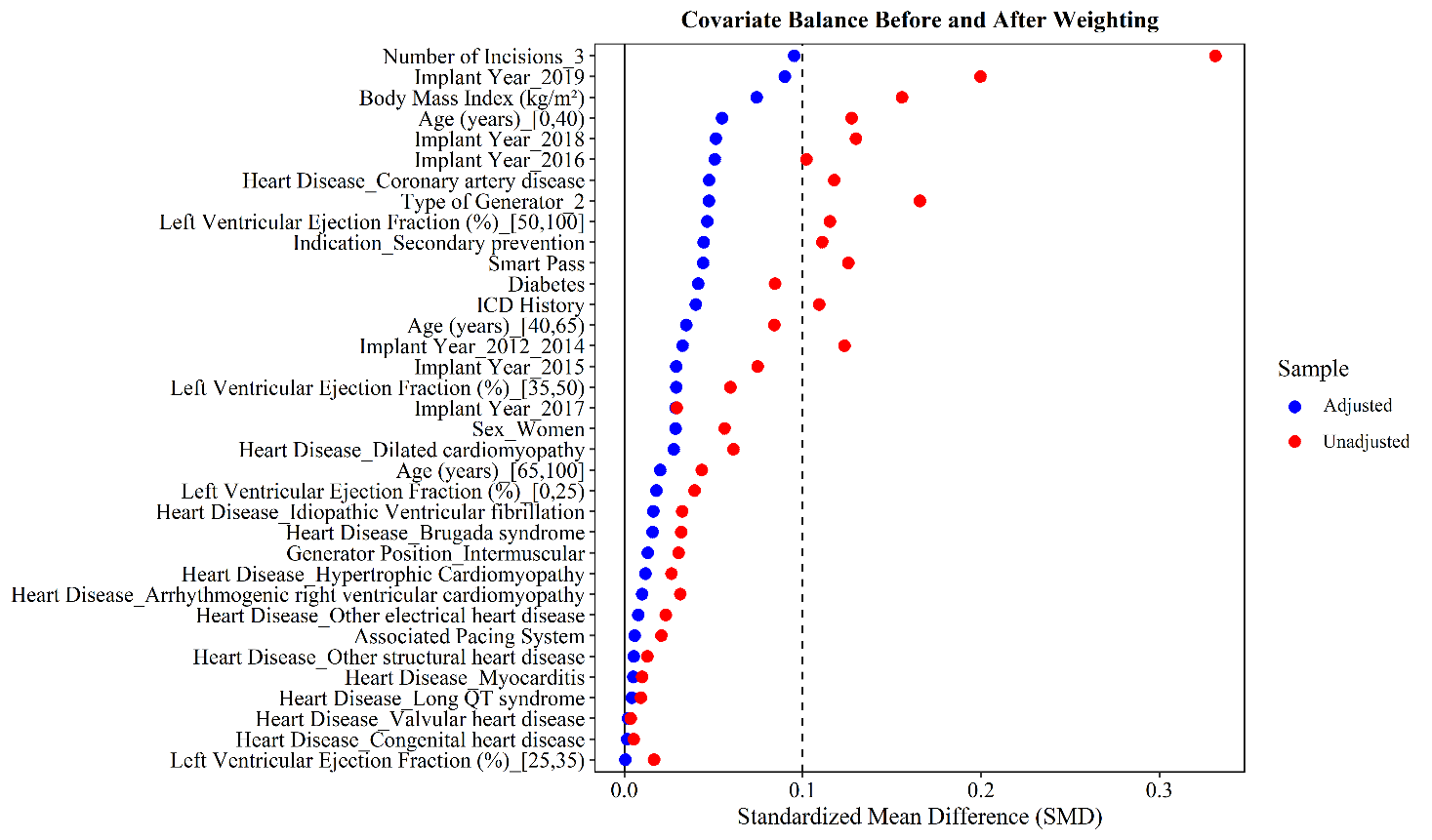
*

**Supplemental Figure 5: Loveplot before and after IPW PS for Complications analysis related to type of non-general anesthesia (NASA vs MAC)**

*
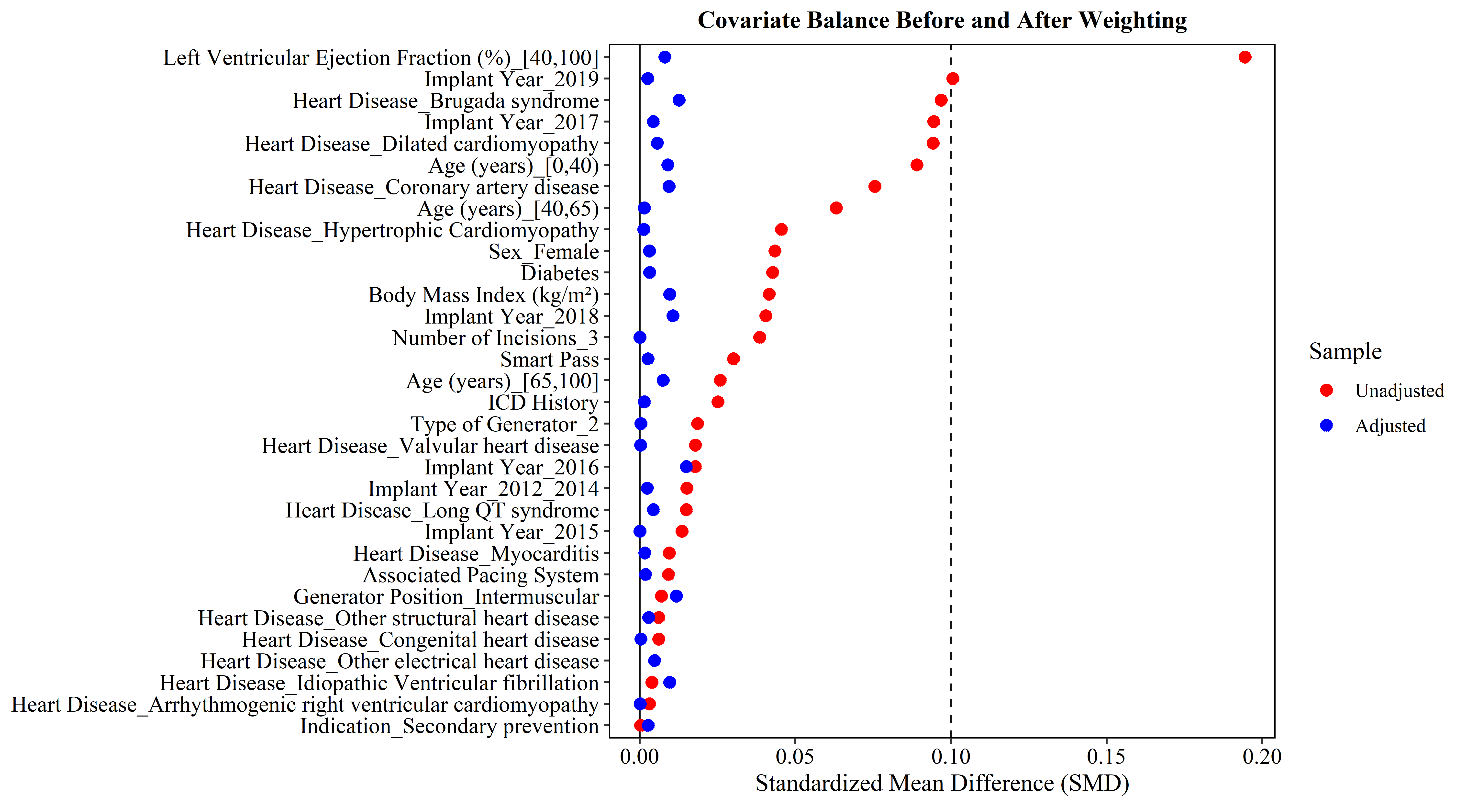
*

**Supplemental Figure 6: Loveplot before and after IPW PS for appropriate Shocks analysis related to type of non-general anesthesia (NASA vs MAC)**

**
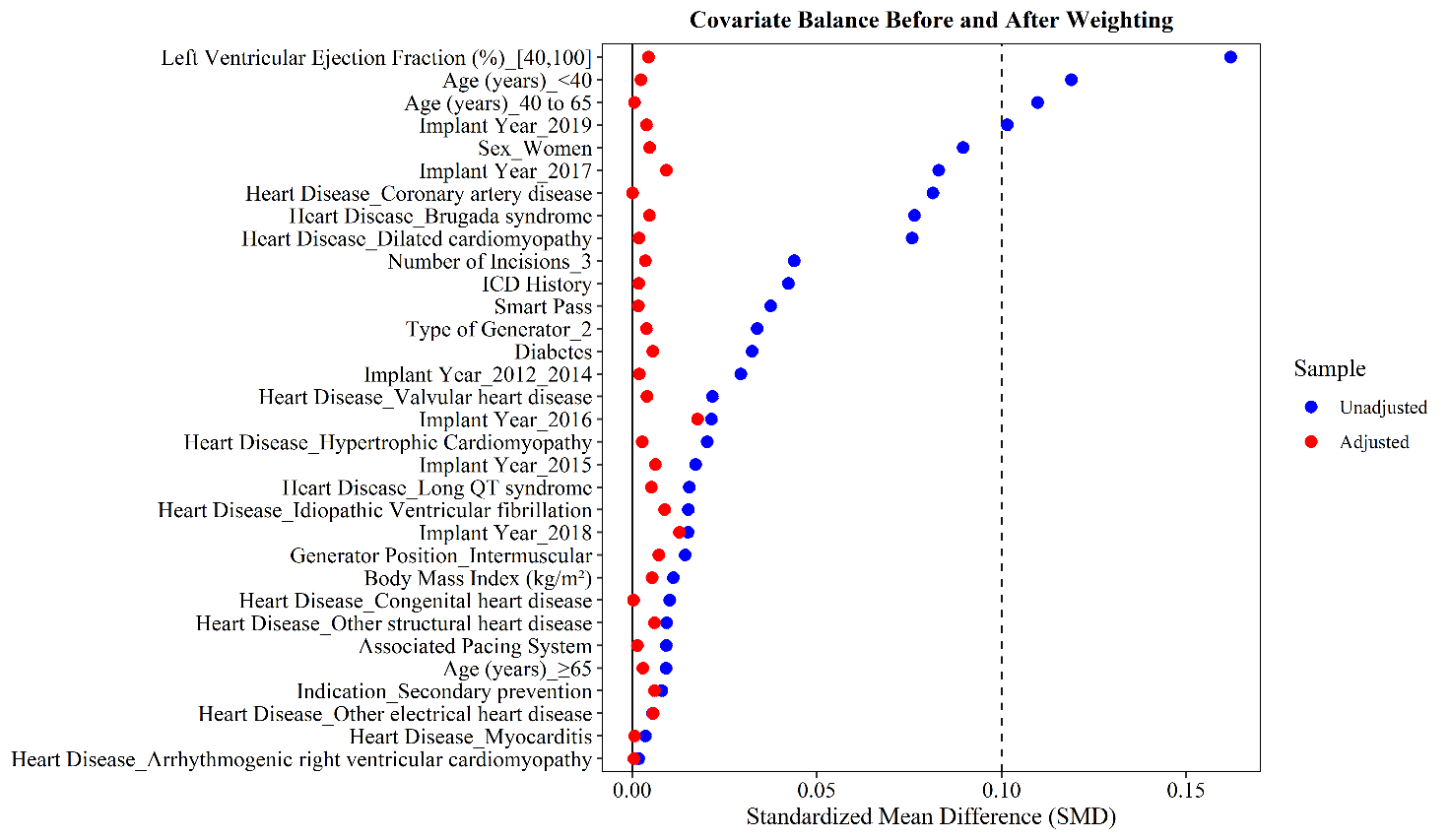
**

**Supplemental Figure 7: Loveplot before and after IPW PS for Inappropriate Shocks analysis related to type of non-general anesthesia (NASA vs MAC)**

*
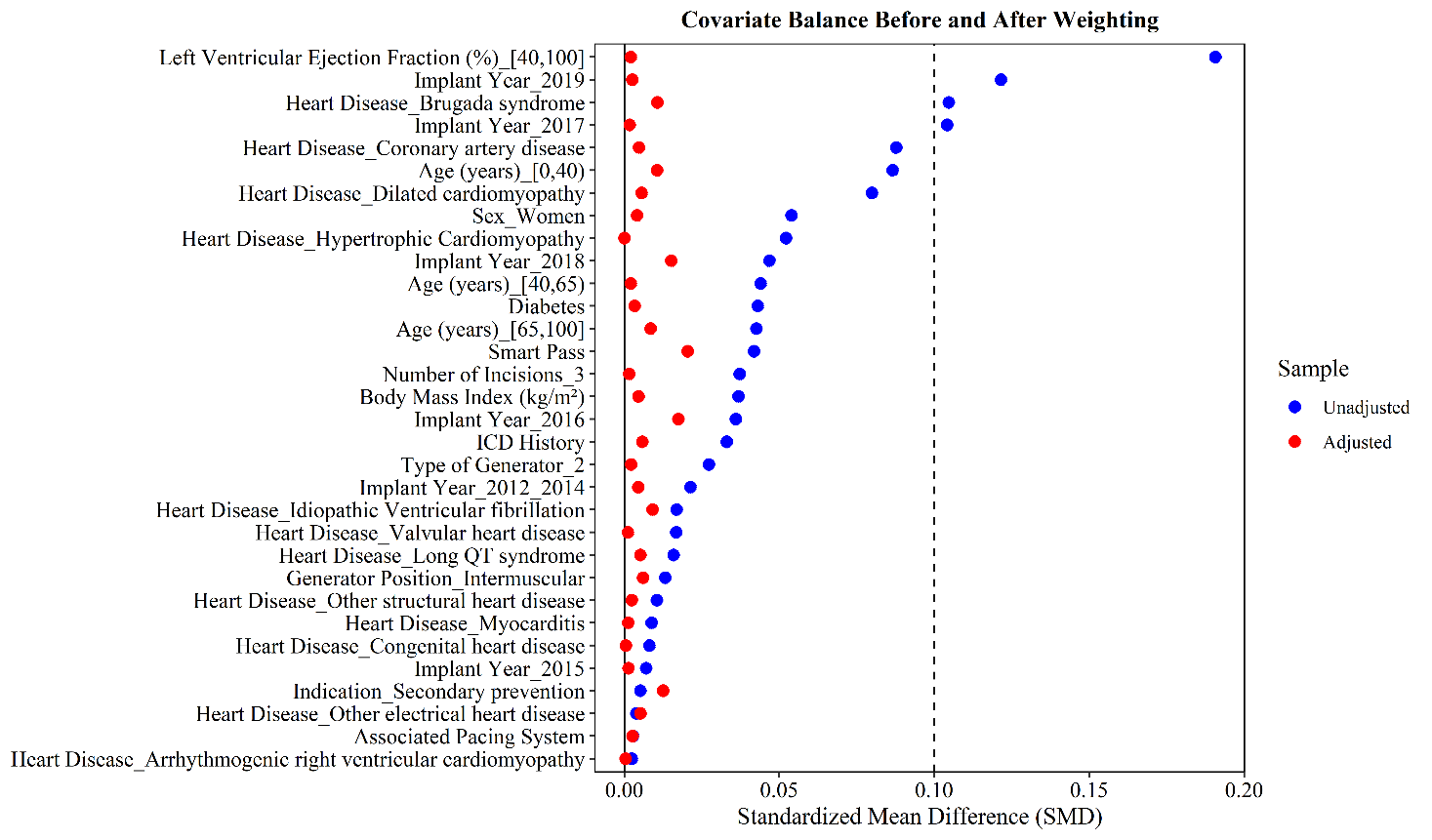
*

**Supplemental Table 1: Baseline Patient Characteristics by Non-General Anesthesia Technique: Monitored Anesthesia Care (MAC) vs. Nurse-Administered Sedation/Analgesia (NASA)**

| **Characteristic*** | **MAC N = 551** | **NASA N = 490** | **p-value** |
| --- | --- | --- | --- |
| **Age** | 52.8 ± 13.1 | 49.7 ± 15.2 | <0.001 |
| **Female Sex** | 102 (18.5%) | 112 (22.9%) | 0.083 |
| **BMI^1^—kg/m²** | 27.0 ± 8.0 | 26.5 ± 8.6 | 0.164 |
| **Diabetes^2^** | 100 (21.1%) | 68 (13.9%) | 0.003 |
| **S-ICD indication** |  |  | 0.992 |
| *Primary prevention* | 369 (67.0%) | 328 (66.9%) |  |
| *Secondary prevention* | 182 (33.0%) | 162 (33.1%) |  |
| **Type of Secondary Prevention** |  |  | 0.027 |
| Cardiac arrest | 130 (71.4%) | 127 (78.4%) |  |
| Sustained ventricular tachycardia | 37 (20.3%) | 32 (19.8%) |  |
| Syncope and inducible VT at EPS | 15 (8.2%) | 3 (1.9%) |  |
| **Underlying heart disease** |  |  | <0.001 |
| *Structural heart disease* | 477 (86.6%) | 363 (74.1%) |  |
| *Electrical heart disease* | 74 (13.4%) | 127 (25.9%) |  |
| **Type of structural heart disease** |  |  |  |
| Ischemic heart disease | 262 (54.9%) | 190 (52.3%) |  |
| Dilated cardiomyopathy | 134 (28.1%) | 73 (20.1%) |  |
| Hypertrophic Cardiomyopathy | 39 (8.18%) | 57 (15.7%) |  |
| Congenital heart disease | 9 (1.9%) | 11 (3.0%) |  |
| Other | 33 (6.9%) | 32 (8.8%) |  |
| **Type of electrical heart disease** |  |  |  |
| Brugada syndrome | 22 (29.7%) | 67 (55.4%) |  |
| CPVT | 0 (0%) | 1 (0.8%) |  |
| Long QT syndrome | 3 (4.05%) | 10 (8.3%) |  |
| Other | 49 (66.2%) | 49 (38.6%) |  |
| **LVEF (%)** | 37.5 ± 15.3 | 44.4 ± 16.4 | <0.001 |
| **History of ICD implantation** | 48 (8.71%) | 55 (11.2%) | 0.175 |
| Reason of ICD removal |  |  | 0.429 |
| *- Infection* | 24 (50.0%) | 29 (52.7%) |  |
| *- Lead failure* | 19 (39.6%) | 24 (43.6%) |  |
| *- Other reason* | 5 (10.4%) | 2 (3.64%) |  |
| Abandoned lead | 14 (29.2%) | 16 (29.1%) | 0.993 |
| **Associated pacing system** | 5 (0.91%) | 9 (1.84%) | 0.194 |
| **Defibrillation testing** | 376 (68.2%) | 267 (54.5%) | <0.001 |
| **Type of generator** |  |  | 0.064 |
| 1st generation 1010 | 10 (1.81%) | 18 (3.67%) |  |
| 2nd and 3rd generation A209/A219 | 541 (98.2%) | 472 (96.3%) |  |
| **Number of incisions** |  |  | <0.001 |
| *2* | 523 (94.9%) | 484 (98.8%) |  |
| *3* | 28 (5.08%) | 6 (1.22%) |  |

*Data are mean ± sd or n (%). ^1^Missing data: 123 for MAC, 24 for NASA. ^2^Missing data: 76 for MAC, 1 for NASA. S-ICD denotes subcutaneous implantable cardioverter defibrillator, LA local anesthesia, GA general anesthesia, VT ventricular tachycardia, EPS electrophysiology study, CPVT Catecholaminergic polymorphic ventricular tachycardia and ICD implantable cardioverter defibrillator.

**Supplemental Table 2**: **Event‐Rate Comparison by Non-General Anesthesia Technique: Monitored Anesthesia Care (MAC) vs. Nurse-Administered Sedation/Analgesia (NASA).**

| **Event** | **Rate/100PY**  **MAC (N=551)** | **Rate/100PY**  **NASA (N=490)** | **Rate Difference** | **p-value** |
| --- | --- | --- | --- | --- |
| **Complications** | **3.87** | **4.73** | **+0.86** | **0.011** |
| **Inappropriate shocks** | **2.70** | **3.12** | **+0.42** | **0.088** |
| **Local complications** | **0.83** | **1.36** | **+0.53** | **0.176** |
| - Infection | 0.20 | 0.60 | +0.40 | 0.051 |
| - Pocket hematoma | 0.54 | 0.35 | -0.19 | 0.464 |
| - Poor wound healing | 0.15 | 0.40 | +0.25 | 0.001 |
| - Other | 0.10 | 0.05 | -0.05 | >0.999 |
| **Lead complications** | **0.20** | **0.20** | **0.00** | **0.853** |
| - Lead dislodgement | 0.10 | 0.10 | 0.00 | >0.999 |
| - Lead fracture | 0.05 | 0.05 | 0.00 | >0.999 |
| - Lead noise | 0.05 | 0.00 | -0.05 | >0.999 |
| - Other | 0.00 | 0.05 | +0.05 | 0.471 |
| **Chronic pain** | **0.20** | **0.25** | **+0.05** | **0.557** |
| **Other complications** | **0.20** | **0.40** | **+0.20** | **0.279** |
| **Complications Requiring Intervention** | **0.78** | **1.71** | **+0.93** | **0.001** |
| **Definite S-ICD removal** | **1.96** | **1.86** | **-0.10** | **0.002** |
| **Pacing need** | **0.59** | **0.55** | **-0.04** | **0.893** |
| **Appropriate shocks** | **2.50** | **1.46** | **-1.04** | **0.034** |
| **Heart transplant** | **0.64** | **0.35** | **-0.29** | **0.640** |
| **Death** | **3.83** | **2.07** | **-1.76** | **0.014** |
| - Cardiovascular | 2.06 | 0.96 | -1.10 | 0.045 |
| - Non-Cardiovascular | 0.98 | 0.86 | -0.12 | >0.999 |
| - S-ICD related | 0.05 | 0.00 | -0.05 | >0.999 |
| - Unknown | 0.74 | 0.25 | -0.49 | 0.128 |

Incidence density is in number of event per 100 person-years

Abbreviations: GA, general anesthesia

**Supplemental Table 3. Multivariate analysis with adjusted HRs for NASA Compared to MAC (MAC as reference category)**

|  | **Crude Analysis** | | **Adjusted Analysis** | |
| --- | --- | --- | --- | --- |
| **Overall Complications** | 1.28 (0.95 – 1.72) | 0.109 | 1.26 (0.92 – 1.73) | 0.148 |
| **Inappropriate Shocks** | 1.25 (0.84 – 1.87) | 0.273 | 1.28 (0.83 – 1.99) | 0.268 |
| **Appropriate Shocks** | 0.89 (0.53 – 1.51) | 0.669 | 0.84 (0.52 – 1.36) | 0.477 |

**Supplemental Table 4. Baseline characteristics of patients lost to follow-up versus retained (MAR assumption)**

| **Characteristic***^1^* | **Not Loss to follow-up** N = 3,584 | **Loss to follow-up** N = 446 | **p value** | **SMD** |
| --- | --- | --- | --- | --- |
| **Age** | 50.1(14.9) | 47.8(16.1) | 0.008 | 0.153 |
| **Female Sex** | 1,048 (23.4%) | 100 (22.4%) | 0.640 | 0.023 |
| **BMI** | 26.5(6.4) | 25.4(4.7) | <0.001 | 0.176 |
| *Unknown* | 787 | 40 |  |  |
| **diabetes** | 640 (16.2%) | 37 (8.37%) | <0.001 | 0.217 |
| *Unknown* | 529 | 4 |  |  |
| **S-ICD indication** |  |  | <0.001 | 0.192 |
| *Primary prevention* | 2,874 (64.2%) | 245 (54.9%) |  |  |
| *Secondary prevention* | 1,604 (35.8%) | 201 (45.1%) |  |  |
| **Underlying heart disease** |  |  | <0.001 | 0.196 |
| *Structural heart disease* | 3,535 (78.9%) | 316 (70.9%) |  |  |
| *Electrical heart disease* | 943 (21.1%) | 130 (29.1%) |  |  |
| **Left ventricular ejection fraction (%)** | 42.0(16.8) | 46.0(16.5) | <0.001 | 0.238 |
| **History of ICD implantation** | 590 (13.2%) | 61 (13.7%) | 0.766 | 0.015 |
| **Associated pacing system** | 80 (1.79%) | 2 (0.45%) | 0.035 | 0.105 |
| **General anesthesia** | 3,554 (79.4%) | 329 (73.8%) | 0.006 | 0.137 |
| **Type of generator** |  |  | 0.019 | 0.165 |
| *1st generation 1010* | 274 (6.12%) | 40 (8.97%) |  |  |
| *2nd and 3rd generation A209/A219* | 4,202 (93.9%) | 406 (91.0%) |  |  |
| *Unknown* | 2 | 0 |  |  |
| **2 incisions** | 4,010 (89.5%) | 410 (91.9%) | 0.114 | 0.078 |
| **Defibrillation testing** | 3,701 (82.6%) | 365 (81.8%) | 0.667 | 0.021 |

**Supplemental Table 5. Subgroup of age >=70 years**

|  | **Crude analysis** | | **IPW Propensity score analysis** | |
| --- | --- | --- | --- | --- |
|  | **HR (95% CI)** | **p-value** | **HR** | **p-value** |
| **Appropriate Shocks** | 1.21 (0.65 – 2.25) | 0.553 | 1.00 (0.50 – 2.02) | 0.990 |
| **Complications** | 1.07 (0.58 – 1.98) | 0.827 | 1.02 (0.55 – 1.88) | 0.960 |
| **Inappropriate Shocks** | 1.17 (0.48 – 2.82) | 0.735 | 1.22 (0.51 – 2.89) | 0.656 |
| **Local Complications** | 1.80 (0.63 – 5.16) | 0.272 | 1.70 (0.60 – 4.84) | 0.317 |
| **Infections** | 0.86 (0.10 – 7.36) | 0.894 | 0.74 (0.09 – 6.38) | 0.781 |
| **Pocket Hematomas** | 1.20 (0.25 – 5.71) | 0.816 | 1.25 (0.28 – 5.69) | 0.770 |
| **Lead Complications** | 0.98 (0.12 – 8.09) | 0.988 | 1.02 (0.13 – 7.84) | 0.982 |
| **Chronic Pains** | 1.43 (0.38 – 5.42) | 0.599 | 1.40 (0.37 – 5.33) | 0.621 |
| **Complications Requiring intervention** | 1.21 (0.33 – 4.46) | 0.774 | 1.14 (0.31 – 4.25) | 0.841 |
| **Definite S-ICD Removals** | 0.85 (0.50 – 1.45) | 0.555 | 0.86 (0.50 – 1.46) | 0.568 |
| **Pacing Needs** | 0.85 (0.32 – 2.29) | 0.755 | 0.84 (0.31 – 2.28) | 0.734 |
| **Deaths** | 0.88 (0.58 – 1.34) | 0.552 | 0.87 (0.56 – 1.33) | 0.516 |
| **Cardiovascular Deaths** | 0.73 (0.37 – 1.43) | 0.355 | 0.72 (0.37 – 1.39) | 0.325 |
| **Non-Cardiovascular Deaths** | 1.24 (0.62 – 2.48) | 0.543 | 1.24 (0.62 – 2.50) | 0.547 |

**Supplemental Table 6. Subgroup of age <70 years**

|  | **Crude analysis** | | **IPW Propensity score analysis** | |
| --- | --- | --- | --- | --- |
|  | **HR (95% CI)** | **p-value** | **HR** | **p-value** |
| **Appropriate Shocks** | 1.15 (0.85 – 1.57) | 0.359 | 1.16 (0.48 – 2.82) | 0.746 |
| **Complications** | 0.90 (0.76 – 1.07) | 0.244 | 0.91 (0.76 – 1.08) | 0.257 |
| **Inappropriate Shocks** | 0.97 (0.77 – 1.23) | 0.791 | 0.98 (0.77 – 1.25) | 0.857 |
| **Local Complications** | 0.84 (0.59 – 1.18) | 0.309 | 0.85 (0.60 – 1.20) | 0.350 |
| **Infections** | 0.64 (0.37 – 1.10) | 0.107 | 0.65 (0.38 – 1.13) | 0.129 |
| **Pocket Hematomas** | 1.20 (0.69 – 2.10) | 0.518 | 1.20 (0.69 – 2.09) | 0.523 |
| **Lead Complications** | 0.57 (0.26 – 1.24) | 0.158 | 0.57 (0.26 – 1.26) | 0.164 |
| **Chronic Pains** | 0.62 (0.27 – 1.46) | 0.276 | 0.60 (0.26 – 1.41) | 0.241 |
| **Complications Requiring intervention** | 0.79 (0.55 – 1.03) | 0.061 | 0.81 (0.56 – 1.10) | 0.102 |
| **Definite S-ICD Removals** | 0.91 (0.64 – 1.30) | 0.601 | 0.92 (0.65 – 1.31) | 0.646 |
| **Pacing Needs** | 0.90 (0.54 – 1.49) | 0.673 | 0.90 (0.54 – 1.49) | 0.681 |
| **Deaths** | 1.18 (0.94 – 1.49) | 0.149 | 1.10 (0.87 – 1.39) | 0.433 |
| **Cardiovascular Deaths** | 1.21 (0.89 – 1.66) | 0.222 | 1.13 (0.82 – 1.55) | 0.463 |
| **Non-Cardiovascular Deaths** | 1.39 (0.90 – 2.17) | 0.141 | 1.38 (0.89 – 2.15) | 0.153 |

**Supplemental Table 7. Subgroup of Males**

|  | **Crude analysis** | | **IPW Propensity score analysis** | |
| --- | --- | --- | --- | --- |
|  | **HR (95% CI)** | **p-value** | **HR** | **p-value** |
| **Appropriate Shocks** | 0.95 (0.71 – 1.28) | 0.755 | 0.85 (0.63 – 1.15) | 0.300 |
| **Complications** | 0.87 (0.72 – 1.05) | 0.154 | 0.88 (0.73 – 1.06) | 0.164 |
| **Inappropriate Shocks** | 0.94 (0.73 – 1.21) | 0.629 | 0.94 (0.73 – 1.22) | 0.653 |
| **Local Complications** | 0.76 (0.51 – 1.14) | 0.184 | 0.77 (0.52 – 1.16) | 0.214 |
| **Infections** | 0.48 (0.24 – 0.97) | 0.041 | 0.49 (0.24 – 0.98) | 0.045 |
| **Pocket Hematomas** | 1.17 (0.64 – 2.14) | 0.602 | 1.16 (0.64 – 2.11) | 0.633 |
| **Lead Complications** | 0.69 (0.29 – 1.66) | 0.412 | 0.72 (0.30 – 1.72) | 0.457 |
| **Chronic Pains** | 0.92 (0.38 – 2.22) | 0.855 | 0.90 (0.37 – 2.16) | 0.812 |
| **Complications Requiring intervention** | 0.67 (0.48 – 0.92) | 0.014 | 0.68 (0.49 – 0.93) | 0.018 |
| **Definite S-ICD Removals** | 0.96 (0.69 – 1.33) | 0.788 | 0.96 (0.69 – 1.33) | 0.800 |
| **Pacing Needs** | 0.95 (0.58 – 1.56) | 0.841 | 0.97 (0.59 – 1.60) | 0.910 |
| **Deaths** | 1.13 (0.91 – 1.41) | 0.278 | 1.03 (0.83 – 1.29) | 0.767 |
| **Cardiovascular Deaths** | 1.17 (0.86 – 1.59) | 0.326 | 1.06 (0.78 – 1.45) | 0.690 |
| **Non-Cardiovascular Deaths** | 1.30 (0.85 – 2.00) | 0.227 | 1.30 (0.85 – 2.00) | 0.228 |

**Supplemental Table 8. Subgroup of Females**

|  | **Crude analysis** | | **IPW Propensity score analysis** | |
| --- | --- | --- | --- | --- |
|  | **HR (95% CI)** | **p-value** | **HR** | **p-value** |
| **Appropriate Shocks** | 1.81 (0.82 – 3.89) | 0.127 | 1.64 (0.76 – 3.52) | 0.204 |
| **Complications** | 1.05 (0.74 – 1.49) | 0.787 | 1.06 (0.74 – 1.51) | 0.759 |
| **Inappropriate Shocks** | 1.15 (0.66 – 2.01) | 0.618 | 1.16 (0.66 – 2.02) | 0.606 |
| **Local Complications** | 1.33 (0.75 – 2.38) | 0.332 | 1.37 (0.76 – 2.44) | 0.294 |
| **Infections** | 1.17 (0.51 – 2.71) | 0.713 | 1.25 (0.54 – 2.89) | 0.609 |
| **Pocket Hematomas** | 1.35 (0.44 – 4.12) | 0.598 | 1.38 (0.45 – 4.22) | 0.572 |
| **Lead Complications** | 0.47 (0.11 – 2.01) | 0.312 | 0.49 (0.11 – 2.07) | 0.329 |
| **Chronic Pains** | 0.58 (0.18 – 1.88) | 0.361 | 0.53 (0.16 – 1.76) | 0.302 |
| **Complications Requiring intervention** | 0.83 (0.50 – 1.37) | 0.458 | 0.88 (0.53 – 1.46) | 0.623 |
| **Definite S-ICD Removals** | 0.74 (0.38 – 1.45) | 0.382 | 0.81 (0.41 – 1.58) | 0.530 |
| **Pacing Needs** | 0.85 (0.30 – 2.45) | 0.766 | 0.85 (0.29 – 2.44) | 0.759 |
| **Deaths** | 1.22 (0.74 – 2.02) | 0.433 | 1.10 (0.66 – 1.83) | 0.721 |
| **Cardiovascular Deaths** | 0.94 (0.44 – 2.01) | 0.872 | 1.06 (0.78 – 1.45) | 0.690 |
| **Non-Cardiovascular Deaths** | 2.04 (0.94 – 4.41) | 0.069 | 2.08 (0.96 – 4.50) | 0.063 |

**Supplemental Table 9. Subgroup of LVEF<35**

|  | **Crude analysis** | | **IPW Propensity score analysis** | |
| --- | --- | --- | --- | --- |
|  | **HR (95% CI)** | **p-value** | **HR** | **p-value** |
| **Appropriate Shocks** | 0.75 (0.52 – 1.09) | 0.129 | 0.76 (0.53 – 1.11) | 0.152 |
| **Complications** | 0.91 (0.71 – 1.17) | 0.476 | 0.91 (0.71 – 1.17) | 0.481 |
| **Inappropriate Shocks** | 0.95 (0.66 – 1.35) | 0.759 | 0.93 (0.65 – 1.33) | 0.677 |
| **Local Complications** | 0.64 (0.37 – 1.11) | 0.114 | 0.63 (0.37 – 1.10) | 0.105 |
| **Infections** | 0.53 (0.23 – 1.25) | 0.146 | 0.52 (0.22 – 1.23) | 0.135 |
| **Pocket Hematomas** | 0.73 (0.31 – 1.76) | 0.489 | 0.70 (0.29 – 1.67) | 0.416 |
| **Lead Complications** | 0.63 (0.22 – 1.80) | 0.389 | 0.65 (0.23 – 1.86) | 0.424 |
| **Chronic Pains** | 1.87 (0.72 – 4.84) | 0.199 | 1.78 (0.69 – 4.63) | 0.236 |
| **Complications Requiring intervention** | 0.72 (0.48 – 1.09) | 0.121 | 0.73 (0.48 – 1.10) | 0.129 |
| **Definite S-ICD Removals** | 1.08 (0.75 – 1.56) | 0.686 | 1.08 (0.75 – 1.57) | 0.668 |
| **Pacing Needs** | 1.10 (0.63 – 1.91) | 0.744 | 1.12 (0.64 – 1.94) | 0.699 |
| **Deaths** | 0.83 (0.64 – 1.07) | 0.156 | 0.84 (0.65 – 1.09) | 0.186 |
| **Cardiovascular Deaths** | 0.95 (0.68 – 1.32) | 0.743 | 0.96 (0.68 – 1.34) | 0.795 |
| **Non-Cardiovascular Deaths** | 1.01 (0.62 – 1.68) | 0.956 | 0.98 (0.60 – 1.60) | 0.930 |

**Supplemental Table 10. Subgroup of patients with diabetes**

|  | **Crude analysis** | | **IPW Propensity score analysis** | |
| --- | --- | --- | --- | --- |
|  | **HR (95% CI)** | **p-value** | **HR** | **p-value** |
| **Appropriate Shocks** | 0.84 (0.46 – 1.55) | 0.585 | 0.80 (0.43 – 1.50) | 0.493 |
| **Complications** | 1.28 (0.86 – 1.89) | 0.228 | 1.19 (0.79 – 1.78) | 0.411 |
| **Inappropriate Shocks** | 1.61 (0.94 – 2.77) | 0.085 | 1.54 (0.89 – 2.67) | 0.124 |
| **Local Complications** | 1.22 (0.59 – 2.52) | 0.591 | 1.24 (0.60 – 2.58) | 0.565 |
| **Infections** | 1.41 (0.49 – 4.03) | 0.525 | 1.31 (0.45 – 3.77) | 0.619 |
| **Pocket Hematomas** | 0.91 (0.25 – 3.30) | 0.884 | 1.08 (0.29 – 3.97) | 0.907 |
| **Lead Complications** | – | – | – | – |
| **Chronic Pains** | – | – | – | – |
| **Complications Requiring intervention** | 1.35 (0.71 – 2.56) | 0.358 | 1.41 (0.74 – 2.70) | 0.298 |
| **Definite S-ICD Removals** | 1.50 (0.82 – 2.75) | 0.190 | 1.44 (0.77 – 2.66) | 0.251 |
| **Pacing Needs** | 1.13 (0.48 – 2.65) | 0.783 | 1.05 (0.44 – 2.48) | 0.913 |
| **Deaths** | 0.85 (0.61 – 1.19) | 0.338 | 0.89 (0.64 – 1.26) | 0.523 |
| **Cardiovascular Deaths** | 0.82 (0.53 – 1.29) | 0.394 | 0.85 (0.53 – 1.36) | 0.490 |
| **Non-Cardiovascular Deaths** | 1.23 (0.64 – 2.36) | 0.544 | 1.21 (0.62 – 2.34) | 0.574 |

**Supplemental Table 11: Steering, Scientific and Adjudication HONEST Committees**

**Steering Committee Members**

| Name | Center |
| --- | --- |
| Serge Boveda | Clinique Pasteur, Toulouse |
| Rodrigue Garcia | Centre hospitalier universitaire de Poitiers |
| Fawzi Kerkouri | Centre hospitalier universitaire de Brest |
| Eloi Marijon | Hôpital européen Georges Pompidou, Paris |
| David Perrot | Hôpital européen Georges Pompidou, Paris |

**Scientific Committee Members**

| Name | Center |
| --- | --- |
| Frédéric Anselme | Centre hospitalier universitaire de Rouen |
| Serge Boveda | Clinique Pasteur, Toulouse |
| Michel Chauvin | Institut cardiovasculaire de Strasbourg |
| Pascal Defaye | Centre hospitalier universitaire de Grenoble |
| Rodrigue Garcia | Centre hospitalier universitaire de Poitiers |
| Jérôme Hourdain | Centre hospitalier universitaire de Marseille, hôpital de la Timone |
| Christophe Leclercq | Centre hospitalier universitaire de Rennes |
| Christelle Marquié | Centre hospitalier universitaire de Lille |
| Eloi Marijon | Hôpital européen Georges Pompidou, Paris |
| Pierre Mondoly | Centre hospitalier universitaire de Toulouse |
| Vincent Probst | Centre hospitalier universitaire de Nantes |
| Nicolas Sadoul | Centre hospitalier universitaire de Nancy |

**Adjudication Committee Members**

| Name | Center |
| --- | --- |
| Frédéric Anselme | Centre hospitalier universitaire de Rouen |
| Nicolas Badenco | La Pitié Salpétrière, Paris |
| Pierre Bertrand | Centre hospitalier universitaire de Tours |
| Geraldine Bertaux | Centre hospitalier universitaire de Dijon |
| Hugues Blangy | Centre hospitalier universitaire de Nancy |
| Serge Boveda | Clinique Pasteur, Toulouse |
| Michel Chauvin | Institut cardiovasculaire de Strasbourg |
| Laure Champ-Rigot | Centre hospitalier universitaire de Caen |
| Estelle Gandjbakhch | La Pitié Salpétrière, Paris |
| Rodrigue Garcia | Centre hospitalier universitaire de Poitiers |
| Charles Guenancia | Centre hospitalier universitaire de Dijon |
| Jérôme Hourdain | Centre hospitalier universitaire de Marseille, hôpital de la Timone |
| Peggy Jacon | Centre hospitalier universitaire de Grenoble |
| Jacques Mansourati | Centre hospitalier universitaire de Brest |
| Christelle Marquié | Centre hospitalier universitaire de Lille |
| Eloi Marijon | Hôpital européen Georges Pompidou, Paris |
| Philippe Maury | Centre hospitalier universitaire de Toulouse |
| Pierre Mondoly | Centre hospitalier universitaire de Toulouse |
| Vincent Probst | Centre hospitalier universitaire de Nantes |
| Nicolas Sadoul | Centre hospitalier universitaire de Nancy |
| Jean Marc Sellal | Centre hospitalier universitaire de Nancy |

**Supplemental Table 12: Data quality audit (2023): types and counts of reporting/classification discrepancies by variable (n=97 across 2,966 audited patients**

| *Variable* | *Type of error* | *Number* |
| --- | --- | --- |
| *Associated pacemaker* | *Not reported as yes* | *1* |
| *Appropriate shocks* | *Not reported event* | *13* |
| *Inappropriate shocks* | *Not reported event* | *21* |
| *Infection* | *Not reported event* | *1* |
| *Pocket hematoma* | *Not reported event* | *2* |
| *Early battery depletion* | *Classified as “Generator Change”* | *46* |
| *Chronic Pain* | *Not reported event* | *12* |
| *Definite S-ICD extraction* | *Not reported event* | *1* |

**Supplemental Table 7. Full list of HONEST investigators**

| Last Name | First Name | Institution |
| --- | --- | --- |
| **Doucy** | Alexandre | Centre Hospitalier Saint Quentin |
| **Barake** | Hassan | Centre Hospitalier de Moulins Yzeure |
| **Kneizeh** | Aziz | Centre Hospitalier de Moulins Yzeure |
| **Scarlatti** | Didier | CHU Nice - Nice |
| **Squara** | Fabien | CHU Nice - Nice |
| **Bun** | Sok-Sithikun | CHU Nice - Nice |
| **Durand** | Philippe | Centre Médico-Chirurgical Arnault Tzanck - St Laurent du Var |
| **Dabiri** | Lara | Clinique Saint Georges - Nice |
| **Ricard** | Philippe | Clinique Saint Georges - Nice |
| **Liprandi** | Laurent | Centre Hospitalier de Cannes |
| **Gauthier** | Garret | Centre Hospitalier de Cannes |
| **Frattini** | Folco | Centre Hospitalier d'Antibes - Juan Les Pins |
| **Dagher Hayeck** | Yann | Centre Hospitalier d'Antibes - Juan Les Pins |
| **Al Amoura** | Alaa | Centre Hospitalier de Troyes |
| **Maillier** | Bruno | Centre Hospitalier de Troyes |
| **Gaty** | Denis | Centre Hospitalier de Carcassonne |
| **Marty** | Lilian | Centre Hospitalier Rodez |
| **Benkaci Ali** | Mohammed | Centre Hospitalier Rodez |
| **Claude Deharo** | Jean | Hôpital de la Timone - CHU Marseille |
| **Hourdain** | Jérome | Hôpital de la Timone - CHU Marseille |
| **Maille** | Baptiste | Hôpital de la Timone - CHU Marseille |
| **Peyrol** | Mickael | Hôpital Nord - CHU Marseille |
| **Barraud** | Jérémie | Hôpital Nord - CHU Marseille |
| **Baptiste** | Florian | Hôpital Nord - CHU Marseille |
| **Taieb** | Jerome | Centre Hospitalier d' Aix-En-Provence |
| **Bouet** | Jerome | Centre Hospitalier d' Aix-En-Provence |
| **Macaluso** | Gilles | Hôpital Privé Marseille - Beauregard |
| **Mechulan** | Alexis | Hôpital privé Clairval - Marseille |
| **PREVOT** | Sébastien | Hôpital privé Clairval - Marseille |
| **Bouharaoua** | Ahmed | Hôpital privé Clairval - Marseille |
| **Gitenay** | Edouard | Hôpital Saint Joseph - Marseille |
| **Bars** | Clement | Hôpital Saint Joseph - Marseille |
| **Seitz** | Julien | Hôpital Saint Joseph - Marseille |
| **Champ Rigot** | Laure | CHU de Caen - Caen |
| **Ollitrault** | Pierre | CHU de Caen - Caen |
| **Milliez** | Paul-Ursmar | CHU de Caen - Caen |
| **Pellissier** | Arnaud | CHU de Caen - Caen |
| **Lebon** | Alain | Hôpital privé Saint-Martin - Caen |
| **Gomes** | Sophie | Hôpital privé Saint-Martin - Caen |
| **Damiano** | Pamela | Centre Hospitalier Henri Mondor - Aurillac |
| **Milhem** | Antoine | Centre Hospitalier de La Rochelle |
| **Duplantier** | Cecile | Centre Hospitalier de La Rochelle |
| **Goujeau** | Cyril | Centre Hospitalier Saintonge - Saintes |
| **Heurtebise** | Isabelle | Centre Hospitalier Jacques Cœur - Bourges |
| **Khris** | Lila | Centre Hospitalier de Bastia - Bastia |
| **Guenancia** | Charles | CHU Dijon Bourgogne - Dijon |
| **Bertaux** | Géraldine | CHU Dijon Bourgogne - Dijon |
| **Laurent** | Gabriel | CHU Dijon Bourgogne - Dijon |
| **Sagnard** | Audrey | CHU Dijon Bourgogne - Dijon |
| **Fichot** | Marie | CHU Dijon Bourgogne - Dijon |
| **Bodi** | Sylvain | Centre Hospitalier de Saint Brieuc |
| **Quentin** | Anne | Centre Hospitalier de Saint Brieuc |
| **Litalien** | Jean | Centre Hospitalier de Périgueux |
| **Courtheix** | Mathieu | Centre Hospitalier de Périgueux |
| **Jarnier** | Philippe | Centre Hospitalier de Périgueux |
| **Badoz** | Marc | CHU Besançon |
| **Favoulet** | Baptiste | CHU Besançon |
| **Serzian** | Guillaume | CHU Besançon |
| **Zimmermann** | Hugues | Clinique Saint Vincent - Besancon |
| **Miralles** | Aurélien | Centre Hospitalier de Valence |
| **Pierre Chatel** | Marie | Centre Hospitalier de Valence |
| **Ramiaramanana** | Eric | Centre Hospitalier Eure-Seine - Evreux |
| **Gorka** | Hervé | Centre Hospitalier de Chartres |
| **Moldovan** | Maria | Centre Hospitalier de Chartres |
| **Laure** | Christophe | Centre Hospitalier de Chartres |
| **Mansourati** | Jacques | CHU de Brest |
| **Kerkouri** | Fawzi | CHU de Brest |
| **Mansourati** | Vincent | CHU de Brest |
| **Hager** | Hugo | CHU de Brest |
| **Winum** | Pierre | Hôpital Carémeau - CHU de Nîmes |
| **Roux** | Julien | Hôpital Carémeau - CHU de Nîmes |
| **Bortone** | Agustin | Hôpital Privé Les Franciscaines - Nîmes |
| **Pujadas** | Pénélope | Hôpital Privé Les Franciscaines - Nîmes |
| **Mondoly** | Pierre | CHU de Toulouse |
| **Maury** | Philippe | CHU de Toulouse |
| **Domain** | Guillaume | CHU de Toulouse |
| **Boveda** | Serge | Clinique Pasteur - Toulouse |
| **Combes** | Nicolas | Clinique Pasteur - Toulouse |
| **Combes** | Stéphane | Clinique Pasteur - Toulouse |
| **Cardin** | Christèle | Clinique Pasteur - Toulouse |
| **Albenque** | Jean-Paul | Clinique Pasteur - Toulouse |
| **Cassagneau** | Romain | Clinique Pasteur - Toulouse |
| **Bordachar** | Pierre | CHU de Bordeaux |
| **Ploux** | Sylvain | CHU de Bordeaux |
| **Sacher** | Frédéric | CHU de Bordeaux |
| **Derval** | Nicolas | CHU de Bordeaux |
| **Strik** | Marc | CHU de Bordeaux |
| **Cesari** | Olivier | Clinique Saint Augustin - Bordeaux |
| **El Bouazzaoui** | Rim | Centre Hospitalier Libourne |
| **Zemmoura** | Adlane | Centre Hospitalier Libourne |
| **Cung** | Thien-Tri | Clinique du Parc - Castelnau le Lez - Montpellier |
| **Cransac** | Frederic | Clinique du Millénaire - Montpellier |
| **Clementy** | Nicolas | Clinique du Millénaire - Montpellier |
| **Luc Pasquié** | Jean | CHU de Montpellier |
| **Garnier** | Mathieu | CHU de Montpellier |
| **Behar** | Nathalie | CHU Rennes - Hôpital Pontchaillou |
| **Leclercq** | Christophe | CHU Rennes - Hôpital Pontchaillou |
| **Galand** | Vincent | CHU Rennes - Hôpital Pontchaillou |
| **Martins** | Raphael | CHU Rennes - Hôpital Pontchaillou |
| **Pavin** | Dominique | CHU Rennes - Hôpital Pontchaillou |
| **Victor** | Frédéric | Polyclinique Saint Laurent - Rennes |
| **Pierre** | Bertrand | CHU de Tours |
| **Fauchier** | Laurent | CHU de Tours |
| **Bisson** | Arnaud | CHU de Tours |
| **Zakine** | Cyril | Clinique Saint-Gatien - Tours |
| **Loose** | Christophe | Clinique Saint-Gatien - Tours |
| **Otmani** | Akli | Clinique Saint-Gatien - Tours |
| **Defaye** | Pascal | CHU Grenoble - Alpes |
| **Jacon** | Peggy | CHU Grenoble - Alpes |
| **Carabelli** | Adrien | CHU Grenoble - Alpes |
| **Venier** | Sandrine | CHU Grenoble - Alpes |
| **Petit** | Luc | Clinique Belledonne - Grenoble |
| **Dreyfus** | Xavier | Clinique Belledonne - Grenoble |
| **Moldovan** | Corina | Centre Hospitalier de Mont de Marsan |
| **Da Costa** | Antoine | Centre Hospitalier Universitaire de Saint Etienne |
| **Romeyer-Bouchard** | Cécile | Centre Hospitalier Universitaire de Saint Etienne |
| **Guichard** | Jean-Baptiste | Centre Hospitalier Universitaire de Saint Etienne |
| **Thevenin** | Jerome | Hôpital Privé de la Loire- Saint Etienne |
| **Probst** | Vincent | CHU de Nantes |
| **Gourraud** | Jean-Baptiste | CHU de Nantes |
| **Arnaud** | Marine | CHU de Nantes |
| **Minois** | Damien | CHU de Nantes |
| **Gras** | Daniel | Hôpital privé du Confluent - Nantes |
| **Giraudeau** | Cédric | Clinique Oreliance - Orléans |
| **Moisei** | Radu | Clinique Oreliance - Orléans |
| **Huguet** | René-Gabriel | Clinique Oreliance - Orléans |
| **Rischard** | Julien | Clinique Oreliance - Orléans |
| **Anys** | Soraya | Clinique Oreliance - Orléans |
| **Monteil** | Benjamin | Clinique Esquirol Saint Hilaire - Agen |
| **Le Page** | Sophie | CHU Angers |
| **Ben Kilani** | Mouna | CHU Angers |
| **Treguer** | Frederic | Clinique St-Joseph - Trélazé - Angers |
| **Merheb** | Michel | Clinique St-Joseph - Trélazé - Angers |
| **Chabert** | Jean-Pierre | CHU Reims |
| **Lesaffre** | Francois | CHU Reims |
| **Espinosa** | Madeline | CHU Reims |
| **Luconi** | Nicolas | CHU Reims |
| **Villemin** | Thibault | Polyclinique Reims-Bezannes-Courlancy |
| **Sandras** | Raphael | Polyclinique Reims-Bezannes-Courlancy |
| **Bel Hadj** | Karim | Clinique Louis Pasteur Essey-lès-Nancy |
| **Schwartz** | Jerome | Clinique Louis Pasteur Essey-lès-Nancy |
| **Olivier** | Arnaud | Clinique Louis Pasteur Essey-lès-Nancy |
| **Beurrier** | Daniel | Clinique Ambroise Paré - Nancy |
| **Zinzius** | Pierre-Yves | Clinique Ambroise Paré - Nancy |
| **Sadoul** | Nicolas | CHU de Nancy |
| **Blangy** | Hugues | CHU de Nancy |
| **Freysz** | Luc | CHU de Nancy |
| **De Chillou** | Christian | CHU de Nancy |
| **Evain** | Stéphane | Centre hospitalier Bretagne Atlantique - Vannes |
| **Rendu** | Eric | Centre hospitalier Bretagne Atlantique - Vannes |
| **Khattar** | Pierre | Groupe Hospitalier Bretagne Sud - Hôpital du Scorff - Lorient |
| **Zanutto** | Aude | CHR Mercy - Metz |
| **Mielczarek** | Marc | CHR Mercy - Metz |
| **Bertrand** | Julien | CHR Mercy - Metz |
| **Becker** | Mathieu | CHR Mercy - Metz |
| **Houriez** | Pierre | Clinique Claude Bernard - Metz |
| **Guyomard** | Yves | Hôpital Saint Philibert - Lille - GHICL |
| **Menet** | Aymeric | Hôpital Saint Philibert - Lille - GHICL |
| **Brimont** | Olivier | Polyclinique Vauban - Valencienne |
| **Bauley** | Karine | Centre Hospitalier de Roubaix |
| **Dennetiere** | Stephane | Centre Hospitalier de Roubaix |
| **Marquié** | Christelle | CHU de Lille |
| **Potelle** | Charlotte | CHU de Lille |
| **Klug** | Didier | CHU de Lille |
| **Sellier** | Romain | Centre Hospitalier de Valenciennes |
| **Forelle** | Laura | Centre Hospitalier de Valenciennes |
| **Mizon-Gérard** | Frédérique | Hôpital privé Le Bois - Lille Metropole |
| **Vaksmann** | Arthur | Hôpital privé Le Bois - Lille Metropole |
| **Elmkies** | Frederic | Centre Hospitalier de Compiègne |
| **Zerah** | Thierry | Centre Hospitalier de Compiègne |
| **Verbrugge** | Eric | Centre Hospitalier de Boulogne sur Mer |
| **Guiot** | Aurélie | Hôpital privé Bois Bernard - Lens |
| **Poueymidanette** | Marc | Hôpital privé Bois Bernard - Lens |
| **Marcant** | Emilie | Centre Médical Chirurgical Obstétrical de la Côte d'Opale - Boulogne Sur Mer |
| **Vanesson** | Claire | Centre Hospitalier de Lens |
| **Hus** | Thibault | Centre Hospitalier de Lens |
| **Eschalier** | Romain | CHU Clermont- Ferrand |
| **Jean** | Frederic | CHU Clermont- Ferrand |
| **Massoullié** | Grégoire | CHU Clermont- Ferrand |
| **Saludas** | Yannick | Pôle Santé République - Clermont- Ferrand |
| **Philippot** | François | Pôle Santé République - Clermont- Ferrand |
| **Roux** | Antoine | Pôle Santé République - Clermont- Ferrand |
| **de Guillebon** | Maxime | Centre Hospitalier de Pau |
| **Bader** | Hugues | Centre Hospitalier de Pau |
| **Gaillard** | Prune | Centre Hospitalier de Pau |
| **Couderc** | Philippe | Clinique Cardiologique D'Aressy |
| **Hebrard** | Aurelien | Clinique Cardiologique D'Aressy |
| **Klotz** | Nicolas | GCS Cardiologie - Bayonne |
| **Laborderie** | Julien | GCS Cardiologie - Bayonne |
| **Lerecouvreux** | Michel | GCS Cardiologie - Bayonne |
| **Demasles** | Christian | Centre Hospitalier de Bigorre - Tarbes |
| **Pripon** | Sorin | Centre Hospitalier de Bigorre - Tarbes |
| **Voglimacci** | Michel | Polyclinique de l'Ormeau - Tarbes |
| **Celse** | Dominique | Polyclinique de l'Ormeau - Tarbes |
| **Lagrange** | Philippe | Clinique Saint Pierre - Perpignan |
| **khoueiry** | Ziad | Clinique Saint Pierre - Perpignan |
| **Sultan** | Pierre | Centre Hospitalier de Perpignan |
| **Nadji** | Georges | Centre Hospitalier de Perpignan |
| **Steinbach** | Mathieu | Centre Hospitalier de Haguenau (Est France) |
| **Bufflerin** | Sebastien | Centre Hospitalier de Haguenau (Est France) |
| **Chauvin** | Michel | Clinique de l'Orangerie - Strasbourg |
| **Schatz** | Alexandre | CHU de Strasbourg |
| **Jesel** | Laurence | CHU de Strasbourg |
| **Pynn** | Sophie | GHCA - Colmar |
| **Bellmont** | Sandrine | GHCA - Colmar |
| **Levy** | Jacques | Centre Hospitalier - Mulhouse - GHRMSA |
| **Le Bouar** | Ronan | Centre Hospitalier - Mulhouse - GHRMSA |
| **Schiau** | Serban | Centre Hospitalier - Mulhouse - GHRMSA |
| **Diene** | Lucien | Centre Hospitalier - Mulhouse - GHRMSA |
| **Bessiere** | Francis | Hospices Civils de Lyon - GH Est-Hôpital Louis Pradel - CHU |
| **Dulac** | Arnaud | Hospices Civils de Lyon - GH Est-Hôpital Louis Pradel - CHU |
| **Chevalier** | Philippe | Hospices Civils de Lyon - GH Est-Hôpital Louis Pradel - CHU |
| **Gardey** | Kevin | Hospices Civils de Lyon - GH Est-Hôpital Louis Pradel - CHU |
| **Durand** | Cyril | Clinique de l'Infirmerie Protestante de Lyon |
| **Durand Dubief** | Alexis | Clinique de l'Infirmerie Protestante de Lyon |
| **Brahic** | Hugo | Clinique de l'Infirmerie Protestante de Lyon |
| **Poty** | Hervé | Clinique de l'Infirmerie Protestante de Lyon |
| **Gal** | Benjamin | Centre Hospitalier Saint Joseph Saint Luc - Lyon |
| **Pineau** | Julien | Centre Hospitalier Saint Joseph Saint Luc - Lyon |
| **Chauveau** | Samuel | Centre Hospitalier Saint Joseph Saint Luc - Lyon |
| **Garrier** | Olivier | Clinique du Tonkin - Lyon - Villeurbane |
| **Attali** | Michael | Polyclinique Lyon-Nord - Rillieux |
| **Fareh** | Samir | Hôpital de la Croix-Rousse - Lyon |
| **Montoy** | Mathieu | Hôpital de la Croix-Rousse - Lyon |
| **Lantelme** | Pierre | Hôpital de la Croix-Rousse - Lyon |
| **Charles** | Paul | Hôpital de la Croix-Rousse - Lyon |
| **Nguyen** | Cédric | Centre Hospitalier Chalon sur Saône |
| **Amelot** | Mathieu | Centre Hospitalier du Mans |
| **Poret** | Philippe | Pôle Santé Sud - Le Mans |
| **Amirault** | Jean-Christophe | Pôle Santé Sud - Le Mans |
| **Bacquelin** | Raoul | Centre Hospitalier de Chambéry |
| **Frey** | Pierre | Centre Hospitalier Annecy Genevois |
| **Irles** | Didier | Centre Hospitalier Annecy Genevois |
| **Dompnier** | Antoine | Centre Hospitalier Annecy Genevois |
| **Akret** | Chrystelle | Centre Hospitalier Annecy Genevois |
| **Siméon** | Edouard | Institut Mutualiste Montsouris |
| **Villejoubert** | Olivier | Institut Mutualiste Montsouris |
| **Mignot** | Nicolas | Institut Mutualiste Montsouris |
| **Jorrot** | Pierre | Institut Mutualiste Montsouris |
| **Mouhoub** | Yamina | Hôpital St. Joseph |
| **Ovart** | Lionel | Hôpital St. Joseph |
| **Ollitrault** | Jacky | Clinique Alleray Labrouste |
| **Amet** | Denis | Clinique Alleray Labrouste |
| **Perrot** | David | HEGP |
| **Varlet** | Emilie | HEGP |
| **Baudinaud** | Pierre | HEGP |
| **Jouven** | Xavier | HEGP |
| **Philibert** | Séverine | HEGP |
| **Pinon** | Pauline | HEGP |
| **Chalbia** | Tej | HEGP |
| **Waldmann** | Victor | HEGP |
| **Marijon** | Eloi | HEGP |
| **Badenco** | Nicolas | La Pitié Salpétrière |
| **Gandjbakhch** | Estelle | La Pitié Salpétrière |
| **Duthoit** | Guillaume | La Pitié Salpétrière |
| **Laredo** | Mikael | La Pitié Salpétrière |
| **Waintraub** | Xavier | La Pitié Salpétrière |
| **Messali** | Anne | Hôpital Bichat |
| **Algalarrondo** | Vincent | Hôpital Bichat |
| **Extramiana** | Fabrice | Hôpital Bichat |
| **Waldmann** | Victor | Hôpital Necker |
| **Bonnet** | Damien | Hôpital Necker |
| **Godin** | Benedicte | CHU de Rouen |
| **Anselme** | Frederic | CHU de Rouen |
| **Savouré** | Arnaud | CHU de Rouen |
| **Chaumont** | Corentin | CHU de Rouen |
| **Auquier** | Nathanael | Groupe Hospitalier du Havre |
| **Elena** | Popescu | Groupe Hospitalier du Havre |
| **Le Franc** | Pierre | Clinique Saint-Hilaire - Rouen |
| **Bouchinet** | Fanny | Clinique Saint-Hilaire - Rouen |
| **Moini** | Cyrus | Clinique Les Fontaines - Melun |
| **Lefoulon** | Audrey | Clinique Les Fontaines - Melun |
| **Belhameche** | Mohamed | Grand Hôpital de l'Est Francilien - Marne-La-Vallée |
| **Sioua** | Sana | Grand Hôpital de l'Est Francilien - Marne-La-Vallée |
| **Bouzeman** | Abdeslam | Hôpital privé de Parly 2 |
| **Bertrand** | Cathy | Hôpital privé de Parly 2 |
| **Halimi** | Franck | Hôpital privé de Parly 2 |
| **Chastre** | Thomas | CHI de Poissy - Saint Germain en Laye |
| **Belkir** | Khadidja | CHI de Poissy - Saint Germain en Laye |
| **Gdalia** | Raphael | CHI de Poissy - Saint Germain en Laye |
| **Amet** | Denis | CHI de Poissy - Saint Germain en Laye |
| **Hermida** | Alexis | CHU Amiens |
| **Otmani** | Akli | CHU Amiens |
| **Kubala** | Maciej | CHU Amiens |
| **Traulle** | Sarah | SAS Cardiologie et Urgences - Amiens |
| **Raguin** | Denis | SAS Cardiologie et Urgences - Amiens |
| **Blaye-Felice** | Marie | Centre Hospitalier d'Albi |
| **Rumeau** | Philippe | Centre Hospitalier d'Albi |
| **Chavernac** | Pascal | Centre Hospitalier Intercommunal Castres-Mazamet |
| **Pouche** | Marion | Centre Hospitalier Intercommunal Castres-Mazamet |
| **El Hajjaji** | Nouredine | Centre Hospitalier de Montauban |
| **Bastard** | Emilie | Polyclinique Les Fleurs - Toulon |
| **Lecardonnel** | Isabelle | Centre Hospitalier Toulon |
| **Lakhal-Ben Larbi** | Essia | Centre Hospitalier Toulon |
| **Cellarier** | Gilles | Hôpital d'instruction des armées Sainte-Anne - Toulon |
| **Demoulin** | Raphaël | Hôpital d'instruction des armées Sainte-Anne - Toulon |
| **Barthez** | Olivier | Centre Hospitalier d'Avignon |
| **Paul Faugier** | Jean | Centre Hospitalier d'Avignon |
| **Cheggour** | Saida | Centre Hospitalier d'Avignon |
| **Hager** | François-Xavier | Clinique Rhône Durance - Avignon |
| **Ortuno** | Frédéric | Clinique Rhône Durance - Avignon |
| **Billon** | Olivier | Centre Hospitalier Départemental Vendée - La Roche-sur-Yon |
| **Garcia** | Rodrigue | CHU de Poitiers |
| **Degand** | Bruno | CHU de Poitiers |
| **Le Gal** | François | CHU de Poitiers |
| **Guy Moyat** | Benoit | CHU de Limoges |
| **Jourda** | François | Centre Hospitalier d’Auxerre |
| **Mourot** | Stéphane | Centre Hospitalier d’Auxerre |
| **Fouché** | Renaud | Hôpital Nord Franche-Comté |
| **Horvilleur** | Jerome | Institut Jacques Cartier - Massy |
| **Fiorina** | Laurent | Institut Jacques Cartier - Massy |
| **Lacotte** | Jerome | Institut Jacques Cartier - Massy |
| **Salerno** | Fiorella | Institut Jacques Cartier - Massy |
| **Younsi** | Salem | Institut Jacques Cartier - Massy |
| **Ait Said** | Mina | Institut Jacques Cartier - Massy |
| **Manenti** | Vladimir | Institut Jacques Cartier - Massy |
| **Mahfoud** | Mohanad | Centre Hospitalier Sud Francilien - Corbeil-Essones |
| **Jacques** | Monteau | Centre Hospitalier Sud Francilien - Corbeil-Essones |
| **Manenti** | Vladimir | Hôpital privé Claude Galien - Quincy-sous-Sénart |
| **Bleinc** | Dominique | Hôpital privé Claude Galien - Quincy-sous-Sénart |
| **Alonso** | Christine | Clinique Ambroise Paré - Neuilly-sur-Seine |
| **Lazarus** | Arnaud | Clinique Ambroise Paré - Neuilly-sur-Seine |
| **Moubarak** | Ghassan | Clinique Ambroise Paré - Neuilly-sur-Seine |
| **Thomas** | Olivier | Clinique Ambroise Paré - Neuilly-sur-Seine |
| **Sharifzadehgan** | Ardalan | Clinique Ambroise Paré - Neuilly-sur-Seine |
| **Zhao** | Alexandre | Clinique Ambroise Paré - Neuilly-sur-Seine |
| **Juin** | Christophe | Hôpital Antoine-Béclère - Clamart |
| **Kanczuga** | Vincent | Hôpital d'Instruction des Armées - Percy |
| **Broustet** | Henri | Hôpital d'Instruction des Armées - Percy |
| **Combes** | Nicolas | Hôpital Marie Lannelongue - Le Plessis Robinson |
| **Maltret** | Alice | Hôpital Marie Lannelongue - Le Plessis Robinson |
| **Benounane** | Abdelhamid | Centre Cardiologique du Nord - Saint Denis |
| **Copie** | Xavier | Centre Cardiologique du Nord - Saint Denis |
| **Piot** | Olivier | Centre Cardiologique du Nord - Saint Denis |
| **Amara** | Walid | Groupe Hospitalier Intercommunal Le Raincy Montfermeil |
| **Abdou** | Vanessa | Groupe Hospitalier Intercommunal Le Raincy Montfermeil |
| **Monsel** | Fabien | Groupe Hospitalier Intercommunal Le Raincy Montfermeil |
| **Lellouche** | Nicolas | Hôpital Henri Mondor - Créteil |
| **Elbaz** | Nathalie | Hôpital Henri Mondor - Créteil |
| **Rouffiac-Noel** | Segolene | Hôpital Henri Mondor - Créteil |
| **Galidie** | Guillaume | Centre Hospitalier d'Argenteuil - Argenteuil |
| **Nitu** | Dorian | Centre Hospitalier René Dubos - Pontoise |
| **Latcu** | Gabriel | Centre Hospitalier Princesse Grace - Monaco |
| **Enache** | Bogdan | Centre Hospitalier Princesse Grace - Monaco |
| **Hugues** | Nicolas | Centre Cardio-Thoracique de Monaco- Monaco |
| **Lagrenade** | Isabelle | Centre Hospitalier de Basse-Terre - Basse-Terre |
| **Demoniere** | Fabrice | CHU Fort-de-France - Fort-de-France |
| **Jocelyn** | Inamo | CHU Fort-de-France - Fort-de-France |
| **Müssigbrodt** | Andréas | CHU Fort-de-France - Fort-de-France |
| **Geoffroy** | Olivier | CHU de la Réunion - Saint Pierre |
| **Clerici** | Gael | CHU de la Réunion - Saint Pierre |
| **Wiart** | François | CHU de la Réunion - Saint Pierre |
| **Ulmer** | Bruno | Centre hospitalier de la Polynésie française - Papeete |
| **Kabalu** | Guillaume | Centre Hospitalier Territorial- Nouméa |
| **Axler** | Olivier | Centre Hospitalier Territorial- Nouméa |
